# The transcription factor *unc-130*/*FOXD3/4* contributes to the biphasic calcium response required to optimize avoidance behavior

**DOI:** 10.1038/s41598-022-05942-0

**Published:** 2022-02-03

**Authors:** Sayaka Hori, Shohei Mitani

**Affiliations:** Department of Physiology, Tokyo Women’s Medical University School of Medicine, Tokyo, 162-8666 Japan

**Keywords:** Genetics of the nervous system, Neural circuits

## Abstract

The central neural network optimizes avoidance behavior depending on the nociceptive stimulation intensity and is essential for survival. How the property of hub neurons that enables the selection of behaviors is genetically defined is not well understood. We show that the transcription factor *unc-130*, a human *FOXD3/4* ortholog, is required to optimize avoidance behavior depending on stimulus strength in *Caenorhabditis elegans*. *unc-130* is necessary for both ON responses (calcium decreases) and OFF responses (calcium increases) in AIBs, central neurons of avoidance optimization. Ablation of predicted upstream inhibitory neurons reduces the frequency of turn behavior, suggesting that optimization needs both calcium responses. At the molecular level, *unc-130* upregulates the expression of at least three genes: *nca-2*, a homolog of the vertebrate cation leak channel *NALCN*; *glr-1*, an AMPA-type glutamate receptor; and *eat-4*, a hypothetical L-glutamate transmembrane transporter in the central neurons of optimization. *unc-130* shows more limited regulation in optimizing behavior than an *atonal* homolog *lin-32*, and *unc-130* and *lin-32* appear to act in parallel molecular pathways. Our findings suggest that *unc-130* is required for the establishment of some AIB identities to optimize avoidance behavior.

## Introduction

Behavioral optimization has a vital function shared by many species: it enables the central nervous system to take reasonable action in response to environmental information^1^. Optimization of avoidance responses to harmful stimuli plays a critical role in defense, and failure directly leads to life-threatening situations^1^. Most animals choose various avoidance behaviors, such as reflexes, retreats, and U-turns, depending on the situation^2^. The neural circuit that evaluates risk to decide the appropriate behavior is not fully understood because of the complexity of these responses compared to simple all-or-none responses^3^. Thus, elucidating the neural and molecular basis is a great challenge.

The zebrafish (*Danio rerio*) and the nematode *Caenorhabditis elegans* (*C. elegans*) have been used as model animals to elucidate the mechanism at the synaptic level. In zebrafish, two Mauthner cells in the hindbrain directly control the muscles and determine the escape direction^4,5^. Mauthner cells can alter glycine receptor expression in a stimulus-dependent manner in addition to following the genetic and developmental programs^5^. The excitation levels of the Mautner cells vary in response to stimulus intensity, but their association with behavioral changes remains unclear.

Previously, we reported that *C. elegans* primarily uses three types of avoidance behaviors depending on the stimulus intensity: short reversals, long reversals, and omega turns^6^. *C. elegans* has an advantage as a model animal because all synaptic connections are anatomically described^7^. The following question remains: how is information processed, and how does it drive behavioral output at the synaptic level? We aimed to reveal a simple prototypical neural circuit at the synaptic level that optimizes avoidance behavior in *C. elegans* by using a complete neural wiring diagram^7,8^ and genes with human orthologs in OrthoList^9,10^.

As part of a neural circuit, ASH sensory neurons mainly perceive nociceptive stimuli, which is the first step, and excite at least three downstream circuits^8^. In the first circuit, ASHs directly form glutamatergic synapses with AIB interneurons, which increases the probability of omega turns^11^. In the second circuit, ASHs indirectly drive AIB activity through cholinergic AIA inhibitory interneurons^11,12^, but the neurotransmitter receptor between AIAs and AIBs is unidentified. In the last circuit, ASHs form glutamatergic synapses with AVA interneurons, whose ON responses and OFF responses (calcium increases) induce reversals and omega turns^6^. AIBs can also affect the OFF-calcium response in the AVA neurons via chemical synapses. AIBs and AVAs coordinate muscle contractions required for turns via motor/interneurons^7,11,12^. We have reported that a lack of AIB cells or gap junction *innexin-1* (*inx-1*) in AIBs reduces turn frequency but increases reversal frequency. These results suggest that AIBs play a central role in optimization and that electrical synapses are components involved in turning^6^. Calcium imaging using a microfluidic system^13^ has revealed that AIBs show strong calcium induction after a hyperosmotic stimulus following a decrease during stimulation^6^.

AIB neurons develop in two steps. First, transcription factors encoded by proneural genes determine progenitors' neuronal fates, and then the progenitors differentiate into mature neurons with unique properties through the combined activity of several transcription factors^14–17^. Previously, we screened 210 neural transcription factors and found that *abnormal cell LINeage 32*, *lin-32*, a nematode homolog of the proneural gene *atonal/ATOH1*^9,18^, is essential for the optimization of avoidance behaviors via AIB neuron development. *lin-32* mutants lack a number of neural function genes in AIBs^6^, and *lin-32* may contribute to the initiation of neurogenesis rather than the determination of AIB neuron identity. Therefore, transcription factors that establish the unique features of AIBs have been left unexplored.

Here, we report that *unc-130*, an ortholog of the *forkhead transcription factor 3 or 4*, *FOXD3/4*^9,10^, plays an essential role in avoidance optimization, mainly by adjusting turn frequency. *unc-130* null mutants lack both ON responses (calcium decreases) and OFF responses (calcium increases) in AIBs. Inhibitory AIAs affect turn frequency, implying their involvement in AIB ON responses (calcium reductions). OFF responses (calcium increases) in the AVAs directly downstream of the AIBs may depend on the AIBs. We report that *unc-130* upregulates three genes: the putative *Nematode CAlcium channel 2* (*nca-2*)^19^, a homolog of the vertebrate cation leak channel NALCN; *AMPA-type glutamate receptor 1* (*glr-1*)^20,21^; and *eat-4*, a hypothetical L-glutamate transmembrane transporter^22^. However, it does not upregulate *synaptobrevin* (*snb-1*)^23–25^ or electrical synapse *innexin 1* (*inx-1*)^9,10^. Our results suggest that *unc-130* partially determines AIB neuron identity to aid in the optimization of avoidance behaviors.

## Results

### A *FOXD3/4* ortholog, *unc-130*, affects the frequency of turn behavior during avoidance optimization

**Figure 1:**
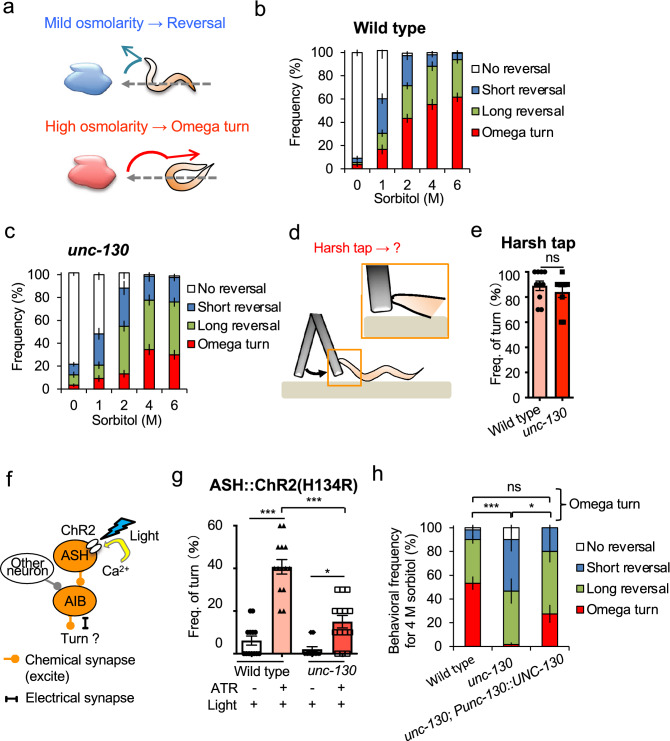
*unc-130* is necessary for turning behavior during optimization of avoidance behavior. (**a**) A schematic of optimization of avoidance behavior in *C. elegans*. During exposure to a mild osmotic stimulus, the animal exhibits a reversal behavior, such as body retraction or backward movement. Strong stimulation induces body bending, causing an omega turn so that the animal can return to its original location^26^. (**b**) In wild-type *C. elegans*, the total frequency of avoidance increased with increasing sorbitol concentration (*P* < 0.001 in 0 M vs. 2–6 M, 1 M vs. 2–4 M, n = 11, 12, 15, 17, 20 (0–6 M, respectively)). In particular, the omega turn frequency was significantly higher under 2–6 M compared to 0 M, under 4 M compared to 1 M, and under 6 M compared to 1 M (*P* < 0.001, *P* < 0.01, *P* < 0.001). (**c**) Nematodes with a null mutation in the *unc-130* had a lower omega turn frequency than wild-type animals (Fig. 1b) (*P* < 0.05 in 1 M and 6 M; n = 12, 12, 14, 20). On the other hand, the total avoidance rate (sum of reversals and omega turns) was similar to that of wild-type animals because of the increase in reversal frequency in all conditions (*P* > 0.05). (**d**) A schematic of the harsh tap assay. A platinum wire placed in the forward direction is lightly applied to the tip of the nematode's nose. **e** *unc-130* mutants showed the omega turn rate comparable to the wild-type animals. *P* = 0.392 (n = 10, 10, t-test). (**f**) Schematic of the analysis method using channelrhodopsin 2 (ChR2(H134)). ChR2 (H134) was selectively expressed in primary nociceptive sensory neurons (ASH sensory neurons)^27^. Exposure to blue light can selectively activate ASH sensory neurons and their downstream neurons. (**g**) Adding all-trans retinoic acid (ATR) and blue light triggered turn behavior. *unc-130* mutants had a significantly lower turn frequency than the wild-type animals. *** indicates *P* < 0.001, * indicates *P* < 0.05 (n = 13, 14, 14, 14). (**h**) Self-promoter-driven UNC-130 expression rescued the decreased turn frequency of *unc-130* mutants. See Fig. S1i–l. *** indicates *P* < 0.001, * indicates *P* < 0.05, ns indicates *P* > 0.05 (n = 6, 6, 4). All statistical analyses were performed by Kruskal–Wallis test with Dunn's multiple comparisons test as a post hoc test. The error bars in the figures represent the ± SEM.

*C. elegans* tends to show reversal behavior in response to mild osmolarity changes but is biased toward omega turns in a manner correlating with stimulus strength (Fig. 1a)^6^. In our previous paper, we mentioned that *unc-130* null mutants exhibited reduced avoidance frequency in response to noxious stimuli^6^. However, it remained unclear whether *unc-130* affected the choice of appropriate behavior or simple locomotion. To clarify this issue, we analyzed the avoidance behavior patterns in response to four gradient osmotic concentrations of noxious stimuli. The results clarified that the *unc-130* mutants were less likely to take omega turns upon hyperosmotic stimulation than wild-type animals (Fig. 1b and c; Fig. S1a–h). Instead, the number of reversals increased in the mutants (Fig. 1c). The critical point is that the *unc-130* mutants had no significant changes in the sum of omega turn and reversal frequencies, suggesting that they can avoid stimuli but not optimize how to do so (Fig. 1b and c; Fig. S1a–h).

Meanwhile, such behavioral phenotype left the possibility that the *unc-130* mutant was unable to make the omega turn because of its uncoordinated movements. We hypothesized that the omega turn might occur by a more intense stimulus. If this were the case, we could conclude that the uncoordinated movements were not the reason for the inability to turn. In the drop test, it was difficult to use a sorbitol solution thicker than 6 M due to the viscosity. Therefore, to investigate whether the *unc-130* mutant can mediate the omega turn as well as the wild-type animals, we established and performed an original harsh tap assay with a platinum wire. In this experiment, we tapped nematodes on the tip of their nose with the platinum wire on NGM plates, and observed their avoidance behavior (Fig. 1d). As a result, the *unc-130* mutant also mediated omega turns in an average of 84.0%, and there was no statistically significant difference compared to the wild-type animals, which mediated omega turns in 89.0% (*P* = 0.392, n = 10, 10, t-test) (Fig. 1e). This data clearly shows that *unc-130* mutants have the physical ability to perform omega turn. Based on the above results, we concluded that *unc-130* affects the behavioral choice. The behavioral phenotype in the null mutants corresponds to “hypoesthesia” in psychological terms^28^.

In the drop test, a sorbitol solution accompanying touch or mechanical stimuli might trigger sensory neurons other than ASHs. To confirm whether the optimization defect in *unc-130* mutants is dependent on noxious stimuli, we observed the behavior induced by selective excitation of ASH sensory neurons using channelrhodopsin-2 (Fig. 1f)^29^. Optogenetic excitation separate from touch or mechanical stimuli reproduced the optimization defect (*P* < 0.001, n = 13, 14, 14, 14, Kruskal–Wallis test with Dunn's multiple comparisons test as a post hoc test) (Fig. 1g), suggesting that *unc-130* optimizes avoidance patterns in response to the strength of the noxious stimuli. UNC-130 driven by its promoter rescued the behavior of the null mutants, indicating that the optimization defect was due to the loss of *unc-130* gene function (*P* < 0.05, n = 6, 6, 4, Kruskal–Wallis test with Dunn's multiple comparisons test as a post hoc test) (Fig. 1h; Figs. S1i–l and S2a–e).

### *unc-130* is necessary for biphasic calcium responses of the central neurons for behavioral optimization

**Figure 2:**
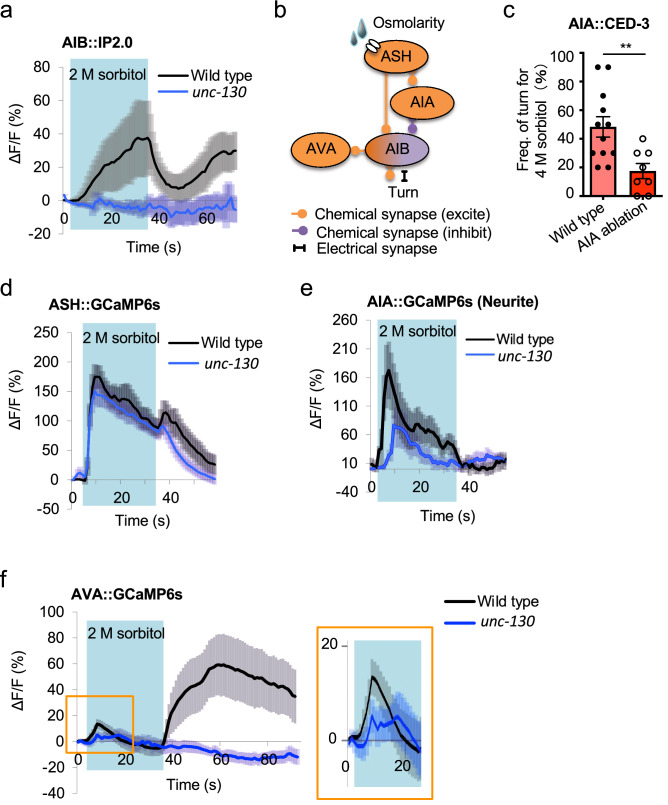
*unc-130* affects both biphasic neural responses of the central neurons for optimization of avoidance behavior. (**a**) Biphasic neural responses in AIBs. IP2.0 is an indicator to monitor the calcium decrease. In wild-type animals, an ON response (calcium decrease) occurred during stimulation (5–35 s; light blue area in the figure) and was followed by an OFF response (calcium increase) immediately after the stimulus (n = 16). In *unc-130* mutants, both of these ON and OFF calcium responses were absent (n = 15). (**b**) Detailed schematic of the downstream neural circuit of ASH sensory neurons. ASH sensory neurons sense osmotic stimuli at receptors and directly excite two types of interneurons (AIBs and AIAs) via glutamate release. Cholinergic AIAs inhibit calcium induction of downstream AIBs. AIBs release glutamate or signal via electrical synapses to excite downstream motor/interneurons, resulting in turn behavior. (**c**) Cell ablation of inhibitory AIA interneurons resulted in reduced turn frequency. ^**^indicates *P* < 0.01 (n = 12, 8, t-test). (**d**) Analysis of calcium responses using G-CaMP6s in ASH sensory neurons (n = 20, 19). (**e**) Calcium imaging of AIA neurites using G-CaMP6s. There was no significant difference in the peak of ON calcium increase in the wild-type animals compared with *unc-130* mutants (*P* = 0.892, n = 21, 23, t-test). (**f**) Calcium imaging of AVA neurons, one of the types of downstream AIB neurons. Wild-type animals showed a small ON calcium increase during stimulation and a large increase after stimulation (n = 20). In the *unc-130* mutants, OFF calcium responses were abolished (n = 19). The area in orange color is enlarged in the right view. The error bars in these figures represent the ± SEM values.

Previously, we showed that AIB neurons are the central neurons for turning and the biphasic response involving a calcium reduction during osmotic stimulation and an increase after stimulation^6^. Considering that *unc-130* expression is relatively restricted in cell lineages including AIB cells (ABplaapa, ABpraapa), during embryogenesis^30^, we hypothesized that the optimization defect of *unc-130* mutants likely resulted from AIB neuronal dysfunction. To test this hypothesis, we attempted to analyze AIB neural responses, including calcium reductions during stimulation, using the calcium indicator inverse-pericam 2.0 (IP2.0), whose fluorescence intensity increases as the calcium concentration decreases^31^. As we expected, the fluorescence intensity of IP2.0 increased during osmotic stimulation and decreased after stimulation in wild-type animals, showing that AIBs received inhibitory input during stimulation and excitatory input after the stimulus was removed (Fig. 2a and Fig. S3a). In contrast, the *unc-130* mutants showed no responses (Fig. 2a). Baseline fluorescence values before correction was close to minimum IP2.0 fluorescence values (Fig. S3bc and c) (*P* = 0.077, n = 14, 18, t-test), implying relatively high resting calcium level in both wild-type and *unc-130* animals (*P* > 0.05, n = 14, 18, t-test). These data suggest that reduced responses in AIB neurons cause the optimization defect in *unc-130* mutants.

Increases in calcium in AIBs induce turning^6,18^, but it remains unclear whether the AIB ON response (calcium decrease) contributes to the turning behavior. In *C. elegans*, ASH sensory neurons indirectly inhibit AIBs via predicted AIA inhibitory interneurons (Fig. 2b)^32^. Therefore, we analyzed whether ablation of AIAs reduces turning. Animals in which AIAs were ablated by caspase CED-3-mediated cell death^33^ exhibited significantly decreased turn frequencies (*P* < 0.01, n = 12, 8, t-test), and AIA-ablated *unc-130* mutants exhibited indifferent turn frequencies (Fig. 2c; Fig. S3d). These results imply that AIA neurons are required for turning, potentially via inhibition of AIBs during the stimulus.

We compared the responses in ASHs using the calcium indicator GCaMP6s^34,35^ to confirm that the other neuron responses in the avoidance circuit remained unaltered, and we found no differences between *unc-130* mutants and wild-type animals (Fig. 2d). This result supports the occurrence of “secondary hypoesthesia” based on a central neuron defect rather than "primary hypoalgesia,” a simple decrease in perception. We also analyzed the calcium responses of the AIAs using GCaMP6s. Since calcium responses in AIAs to noxious osmolarity stimuli have not yet been reported, we started by observing the detailed patterns in wild-type animals. We found increased calcium concentrations in AIA neurites during osmotic stimulation (Fig. 2e; Fig. S3e). In previous studies, calcium imaging has been performed in AIA neurites^36^. One reason is that AIA neurites, but not the cell bodies, show large calcium fluctuations. Fortunately, we were able to observe calcium responses in AIA cell bodies using our original transgenic strain (Fig. S3f). We found that AIA cell bodies show a triphasic pattern: a sustained increase in calcium concentration during stimulation, a decrease for 30 s after the end of stimulation, and a subsequent increase (Fig. S3f). This response pattern appears to correlate more strongly with calcium decrease responses in AIB neurites than with responses in AIA neurites (Fig. S3a, e and f). Considering that the AIA-AIB synapse is a predicted inhibitory circuit, the responses in the AIA cell body might affect the biphasic response in the AIB. Then, we observed the AIA reaction in the *unc-130* mutants and clarified that there was no statistically significant variation in peak ΔF/F amplitude (*P* = 0.892, n = 21, 23, t-test), although the response was slightly dampened in mutants compared within wild-type animals (Fig. 2e).

Finally, to understand the output function of AIBs on the avoidance circuit, we observed the calcium response in AVAs. AVAs receive input from the presynaptic AIB neurons (Fig. 2b). As expected, *unc-130* mutants showed remarkably impaired OFF responses (calcium increases) in AVAs (Fig. 2f). In our previous paper, we discussed how low ON responses (calcium increases) in AVAs during osmotic stimulation might induce reversal behavior independent of AIBs^6^. Since the *unc-130* mutants can perform reversal behavior (Fig. 1c; Fig. S1f and g), we explored whether the AVA ON response (calcium increase) remains in the *unc-130* mutants. The *unc-130* mutants showed an ON response (calcium increase), although it was reduced in size (see the enlarged view of the area in Fig. 2f, inset), consistent with our hypothesis. These results suggest that AIBs are critical neurons for determining appropriate behaviors in response to various stimuli.

The inhibitory synaptic receptors on AIBs have not yet been identified. Since AIAs are cholinergic neurons^37^, we first considered the possibility of the downregulation of inhibitory acetylcholine-gated chloride channels expressed on AIBs in the *unc-130* mutants. We observed the expression patterns of six hypothetical acetylcholine-gated or ligand-gated chloride channels to focus on the inhibitory channels expressed on AIB neurons. In this experiment, we found that all *unc-130* mutant animals expressed *innexin 1* (*inx-1*) on AIBs (n = 25); we used the *inx-1* promoter to mark AIBs and identify AIB-specific gene expression. AIBs expressed *acc-1 (Acetylcholine-gated Chloride Channel 1)*^38^*, lgc-46 (Ligand-Gated ion Channel 46,* predicted to have chloride channel activity*)*, and *lgc-49 (Ligand-Gated ion Channel 49,* predicted to have chloride channel activity*)*^39^ promoter-driven GFP (Fig. S4a). However, AIBs did not express *acc-2*, *acc-3*, or *acc-4 (Acetylcholine-gated Chloride Channel 2, 3,* or *4*, respectively*)*^38^ (Fig. S4b), nor did they express *lgc-47* or *lgc-48* (*Ligand-Gated ion Channel 47* or *48,* respectively, predicted to have chloride channel activity)^10^. We analyzed the turn behavior of deletion mutants of *acc-1*, *lgc-46*, and *lgc-49*, but there were no differences between the single or double mutants and wild-type animals (Fig. S4c and d).

We next assumed a contribution of G protein-coupled acetylcholine receptors. We observed turn behavior in deletion mutants of *goa-1* (*G protein O, alpha subunit 1*; an ortholog of human *GNAO1*^10^), which exhibits G protein-coupled acetylcholine receptor activity^40^. In *C. elegans*, only *goa-1* encodes a member of the mammalian Gi/o class of Gα subunits, and the predicted amino acid sequence of *C. elegans* GOA-1 is over 80% identical to that of mammalian Gαo^40^. Hypothetical *goa-1* null mutants showed a lower turn frequency (13.3 ± 4.41%) than wild-type animals (56.3 ± 5.96) (*P* < 0.001, n = 9, 8, t-test) (Fig. S5a), but the expression levels of *goa-1* promoter-driven GFP in AIBs were comparable in both strains (Fig. S5b and c) (*P* = 0.450, n = 10, 9, t-test). Thus, we concluded that the reduction in turning was independent of the *goa-1* function in AIB neurons. In *C. elegans*, there are three types of muscarinic-type acetylcholine receptors: *gar-1 (G-protein-linked Acetylcholine Receptor 1*)^41^, *gar-2*^42^, and *gar-3*^43^. Although we analyzed the behavior of triple gene mutants^44^, turn frequency was not significantly lower in these animals than in wild-type animals (Fig. S5d) (*P* = 0.226, n = 9, 8, t-test). Consequently, we could not identify the AIA-AIB inhibitory receptors, but the possibility remains that an unknown G-protein coupled acetylcholine receptor participates.

### *unc-130* upregulates the expression of a predicted cation channel

**Figure 3:**
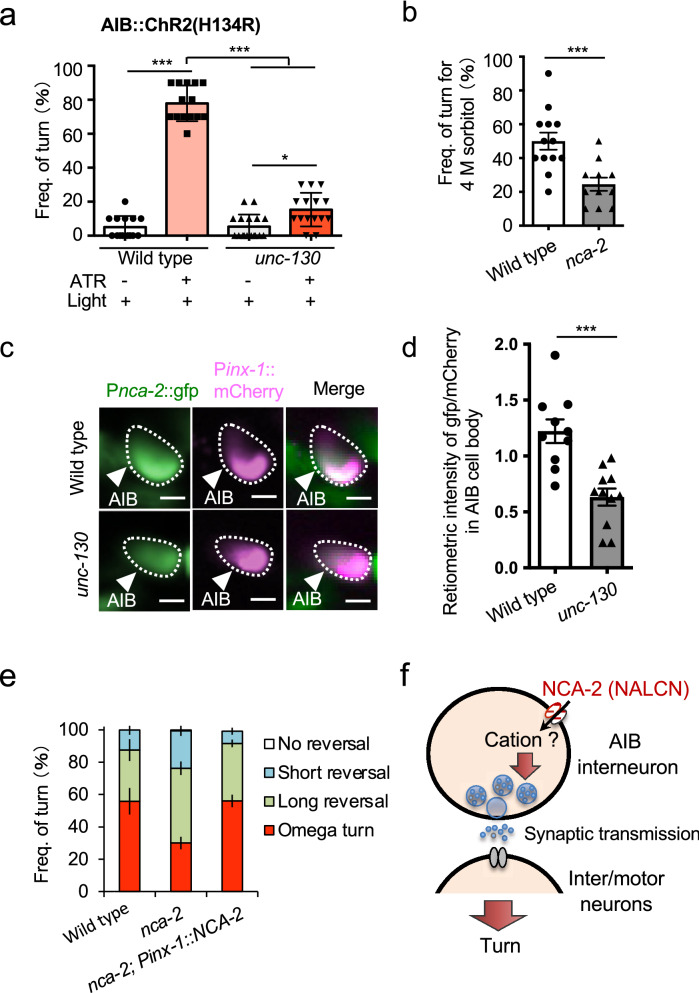
*unc-130* upregulates the expression of putative cation channels in AIB neurons. (**a**) All-trans retinoic acid and blue light induce turning. The turning frequency was 77.9% in the wild type but only 15.3% in *unc-130* mutants. ^***^indicates *P* < 0.001, ^*^indicates *P* < 0.05 (n = 14, 14, 17, 15, Kruskal–Wallis test with Dunn's multiple comparisons test as a post hoc test). (**b**) *nca-2* mutants showed significantly reduced turn frequencies in response to 4 M sorbitol than wild-type animals. ^***^indicates *P* < 0.001 (n = 13, 11, t-test). (**c**) Expression of *nca-2* promoter-driven GFP in AIB cell bodies (arrowheads). *inx-1* promoter-driven mCherry was coexpressed as a cell identification marker for AIBs. *nca-2* showed weaker AIB expression in a typical *unc-130* animal than in a typical wild-type animal. Scale bar = 5 µm. (**d**) The ratiometric analysis of the intensity of *nca-2* promoter-driven GFP expression was significantly lower in AIBs of *unc-130* mutants than in those of wild-type animals. ^***^indicates *P* < 0.001 (n = 10, 11, t-test). (**e**) AIB-selective expression of NCA-2a rescued the decreased turn frequency of *nca-2* mutants. ** indicates *P* < 0.01 (n = 12, 12, 12, one-way ANOVA followed by Tukey’s post hoc test). See Fig. S7a–d. The error bars in these figures represent the ± SEM values.** (f**) Model diagram of the hypothetical function of *nca-2* in AIB. NCA-2 may involve the release of neurotransmitters from synaptic vesicles via cation influx.

*unc-130* mutants showed no stimulus-dependent OFF responses (calcium increases) (Fig. 2a). Therefore, we tested whether the turn behavior could be recovered by cation flux through ChR2(H134R) selectively expressed in AIBs. Contrary to our expectations, turn behavior induction was minor in *unc-130* mutants (Fig. 3a). In optogenetics, cation flux is reported to be milder than that under natural stimulation^45^. Therefore, we proposed three hypotheses: 1. that there is dysfunction in the calcium signaling pathway in AIBs, 2. that there are reductions in presynaptic output from AIBs, and 3. AIBs are developmentally altered in unc-130 mutants.

First, we tested the former. There are eight of nine predicted voltage-gated calcium channel subunits in *C. elegans*^46^. We excluded three genes from our analysis. One of them, *ccb-1* (*Calcium Channel, Beta subunit 1*, predicted to have high voltage-gated calcium channel activity), is not expressed in AIBs^46^. We performed a drop test for mutants of the other seven channels and found that only *nca-2* (*putative Nematode CAlcium channel 2*, a homolog of the vertebrate cation leak channel *NALCN*) mutants showed a lower turn frequency in response to a 4 M sorbitol drop than wild-type animals (Fig. 3b; Fig. S6a and b) (*P* < 0.001, n = 13, 11, t-test). The mutants for the other five channels, *tag-180* (an ortholog of human voltage-gated calcium channel auxiliary subunit alpha 2 delta 2)^47^ (Fig. S6a), *unc-2 (UNCoordinated 2,* a predicted voltage-gated calcium channel*)*^48^, *nca-1* (*putative Nematode CAlcium channel 1*)^19^, *cca-1* (*Calcium Channel Alpha subunit 1*)^49^, *ccb-2* (*Calcium Channel, Beta subunit 2*)^19^, and *unc-36* (*UNCoordinated 36*)^47^, showed no reductions in turn frequency (Fig. S6b). However, it should be noted that *nca-1 (gk9)* and *ccb-2 (ok862)* provide no evidence for null alleles only because they are predicted to have hypothetical reduced expression and partial loss of a domain, respectively (Fig. S13). So we could not completely exclude the possible roles of these channels.

To uncover whether *unc-130* regulates *nca-2* expression in AIBs, we quantified GFP intensity driven by the *nca-2* promoter. The results showed that AIBs expressed *nca-2*, but the *unc-130* mutants showed significantly reduced fluorescence intensity compared with wild-type animals (Fig. 3c and d). The neural subsets expressed *nca-2*. To test whether reduced *nca-2* expression in the AIBs correlates with reduced turn frequency, we performed a rescue experiment with the AIB-selective expression of the NCA-2a protein. The results revealed that the defect in *nca-2* mutants was substantially alleviated such that the mutants recovered an optimization pattern relatively similar to that of wild-type animals (Fig. 3e; Fig. S7). Thus, we conclude that *nca-2* expression in AIBs is necessary for avoidance behavior optimization, suggesting a voltage-gated cation channel or a cation leak channel expression in the AIBs may upregulate synaptic transmission from AIBs to downstream inter-/motor neurons (Fig. 3f).

### *unc-130* upregulates the expression of functional molecules for glutamatergic synapses

**Figure 4:**
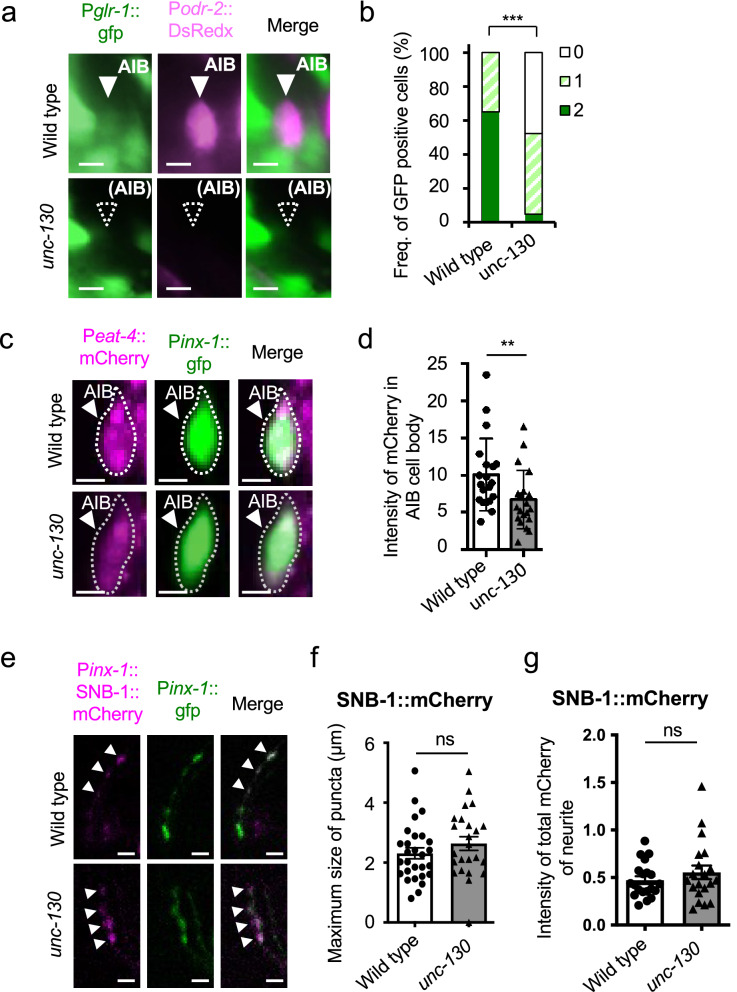
*unc-130* affects the expression of two molecules in glutamate synapses in AIB neurons. (**a**) Expression analysis of *glr-1* promoter-driven GFP. DsRedx driven by the promoter of the olfactory receptor *odr-2* was coexpressed as a cell identification marker for AIBs. *glr-1* was weakly expressed in AIBs in a wild-type animal (arrowhead), while both *glr-1* and *odr-2* were absent in an *unc-130* mutant. Scale bar = 5 µm. (**b**) Frequency of animals expressing GFP in AIB neurons. Because we used the mosaic Ex strain, GFP was expressed in two AIB cells at 65.5% and in one cell at 35.0% in wild-type animals. The frequencies of animals with GFP-positive AIBs were 4.76% for two cells and 47.6% for one cell; 47.6% had no expression among the *unc-130* mutants. ^***^ indicates *P* < 0.001 (n = 20, 21, Fisher’s exact test). (**c**) The expression of mCherry driven by the promoter of the vesicular glutamate transporter *eat-4* was weak in an *unc-130* mutant. Scale bar = 5 µm. (**d**) The intensity of mCherry expression in AIBs of *unc-130* mutants was significantly reduced. ^**^indicates *P* < 0.05 (n = 20, 20, Mann–Whitney test). (**e**) Comparison of synaptic localization patterns. The arrowheads indicate accumulated SNB-1::mCherry in AIB neurites. Scale bar = 5 µm. (**f**) The distribution of the maximum accumulation size of SNB-1::mCherry was not different between wild-type and *unc-130* mutants. “ns” indicates no significant difference (*P* = 0.262, n = 28, 24, t-test). (**g**) The ratiometric analysis of the total intensity of mCherry expression in AIB presynaptic regions was not different between wild-type and *unc-130* mutants. “ns” indicates no significant difference (*P* = 0.326, n = 20, 20, t-test). The error bars in these figures represent the ± SEM values.

Transcription factors frequently determine neural identity by regulating the expression of multiple genes^46^. To determine whether *unc-130* contributes to the excitatory input from upstream neurons to the AIB, we observed *glr-1* (*GLutamate Receptor family (AMPA) 1*) promoter-driven GFP expression. *glr-1* is one of the primary excitatory receptors in AIB neurons^50^. GLR-1 is the primary glutamate receptor between ASHs and AIBs. Among the wild-type animals, 65.0% of individuals expressed GFP in two AIB cells, while 35.0% expressed it in one (Fig. 4a and b). Notably, transgenic *C. elegans* carrying extrachromosomal transgenes frequently display mosaic expression^47^. Among the *unc-130* mutants, only 5.0% of individuals expressed GFP in two AIB cells, while 52.4% expressed it in one (Fig. 4a and b); mutants showed a significant difference compared with the wild-type animals. This result indicates a reduction in excitatory synaptic input from the ASHs. We also tried to use the P*inx-1*-driven markers, but the transgenic strains could not be maintained, so we used P*odr-2* as the marker promoter in this experiment. AIBs are glutamatergic neurons^51^. To determine whether typical glutamatergic synaptic vesicles are formed in AIBs, we analyzed the expression level of *eat-4* (a predicted L-glutamate transmembrane transporter), which fills vesicles with glutamate^37^. We detected a significant reduction in the fluorescence intensity of *eat-4* promoter-driven mCherry in the AIBs of *unc-130* mutants (*P* < 0.05, n = 20, 20, Mann–Whitney test) (Figs.4c and d), suggesting that *unc-130* is required for the release of proper amounts of glutamate. A decrease in the expression of *eat-4* might cause the decrease in turns upon AIB::ChR2 stimulation shown in Fig. 3a.

We suspected that the *unc-130* mutant forms abnormal presynapses. Then, we observed the localization of mCherry-tagged synaptobrevin (SNB-1), a typical presynaptic marker, at synaptic sites. However, both the maximum size and the total fluorescence intensity of SNB-1::mCherry fusion puncta indicated no significant differences between the *unc-130* mutants and wild-type animals (Fig. 4e–g). RAB-3, a component of synaptic vesicles^52^, also showed no differences in accumulation size and fluorescence intensity between the two strains. These results indicate that *unc-130* is required for both the input and output of glutamatergic synaptic transmission at AIB neurons but not for the fundamental function of synapse formation.

### *unc-130* does not alter the expression of electrical synapses in AIBs

**Figure 5:**
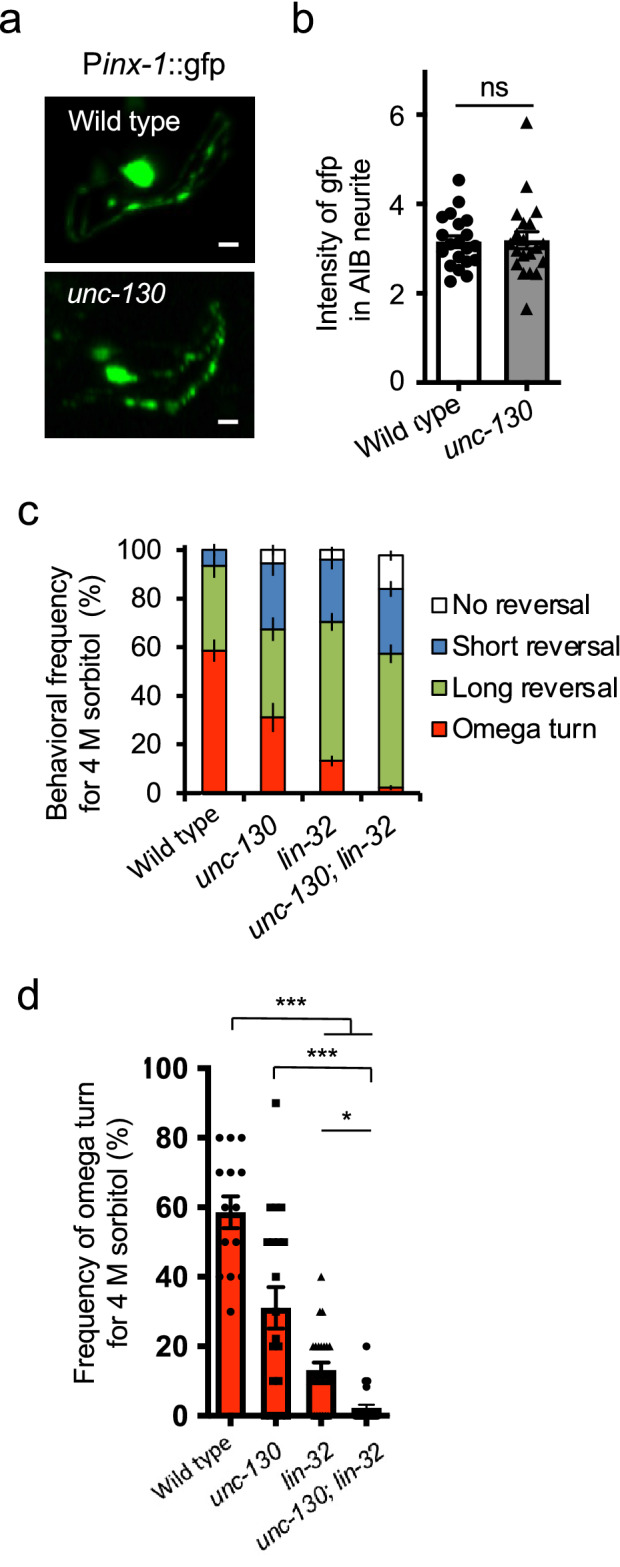
*unc-130* does not regulate electrical synapses in the AIBs. (**a**) Comparison of the expression of *inx-1* promoter-driven GFP. *unc-130* mutants showed similar expression levels. Scale bar = 5 µm. (**b**) There were no differences in the intensity of *inx-1* promoter-driven GFP expression measured in cell bodies between the wild-type animals and the *unc-130* mutants. “ns” indicates no significant difference (*P* = 0.960, n = 20, 20, Mann–Whitney test). (**c**) The double mutants of *unc-130* and *lin-32* almost completely lacked turn behavior (n = 26), whereas *unc-130* and *lin-32* single mutants exhibited moderately lower turn frequencies (n = 19, 25) than wild-type animals (n = 14). Each mutant had an increased reversal frequency instead of exhibiting turning. (**d**) *unc-130* and *lin-32* single mutants (n = 19, 25) showed significantly different turn frequencies than the wild-type animals (n = 14) and the double mutants (n = 26). ^***^indicates *P* < 0.001, ^*^indicates *P* < 0.05 (Kruskal–Wallis test with Dunn’s multiple comparisons test as a post hoc test). The error bars in these figures represent the ± SEM values.

Previously, we reported that *lin-32* upregulates a broad spectrum of genes in AIB neurons, including the dominant electric synapses *inx-1*, and that its mutation causes secondary hypoesthesia similar to that caused by *unc-130* mutation^6^. Therefore, we analyzed the property of *inx-1* positive cells, expression rate, cell morphology, and expression intensity, as one AIB indicator of functional overlap between *unc-130* and *lin-32*. First, in terms of expression rate, *GFP*-positive cells were observed in all *unc-130* mutants (n = 128) (Fig. S8a and b). 116 (90.6%) animals had normal AIB location and morphology, while 12 animals (9.38%) had an ectopic dendrite extending to the tip of the nose (Fig. S8a and b). However, the occurrence rates were not significantly different (*P* = 0.0714, n = 40, 128, Fisher's exact test), and there was no association between the presence of ectopic neurites and turn/reversal frequency (Fig. S8c) (*P* = 0.892, n = 40, 128, Fisher's exact test). They suggest that this morphological is not related to the optimization of avoidance behavior. Next, to determine whether *unc-130* might affect the amount of *inx-1* expression, we quantified the intensity of GFP driven by the *inx-1* promoter in the mutants. The *unc-130* mutants expressed the same amount of *inx-1* as wild-type animals (Figs. 5a and b) (*P* = 0.326, n = 20, 20, Mann–Whitney test), indicating that *unc-130* regulates genes, *inx-1* different from those regulated by *lin-32*. Finally, we performed a drop test on the double mutants to confirm that *unc-130* and *lin-32* act in different molecular pathways. The double mutants exhibited more remarkable optimization defects than the single mutants (Fig. 5c), and the turns almost completely disappeared (Fig. 5d). Meanwhile, the reversal ability remained (Fig. 5b; Fig. S12), suggesting that *unc-130* and *lin-32* regulate parallel molecular pathways for turning. We conclude that *unc-130* impacts behavioral optimization by regulating the characteristic gene expression of calcium dynamics and glutamatergic synapse function, unlike *lin-32*.

*unc-130* starts to be expressed in early embryogenesis but not detected in neurons from the larvae stage onward^53^. In order to clarify whether *unc-130* contributes to AIB development and determination decisions in embryogenesis or gene expression in the adult stage, we examined behavioral rescue in the adult driven *unc-130* by the *inx-1* or *npr-9* promoters, which cause AIB-selective expression from larva onward. As a result, all three or four independent transgenic lines driven UNC-130 from the larva onward could not rescue (Figs. S9 and S10), suggesting that function during embryogenesis is essential. Behavioral optimization defects in *unc-130* adults were not rescued by overexpression of NCA-2a or EAT-4 driven by the *inx-1* promoter (Figs. S10 and S11). Co-transduction with multiple genes in AIB regulated by *unc-130*—at least *nca-2*, *eat-4* and *glr-1* – might be able to rescue behavioral defects.

## Discussion

### *unc-130* regulates the restricted genes for specific neuronal phenotypes to optimize behavior

**Figure 6:**
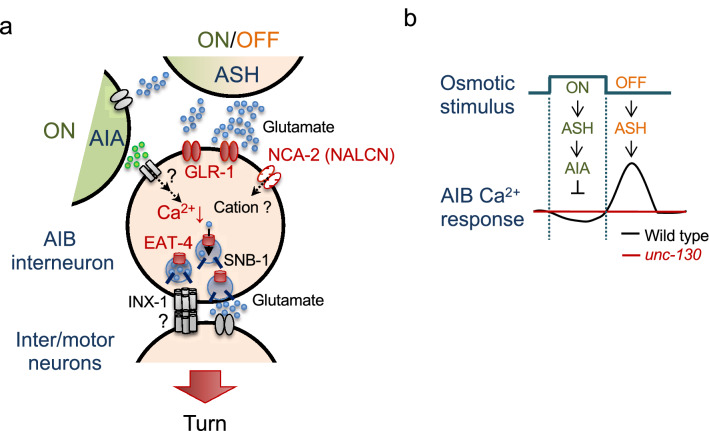
Schematic model of the AIB calcium response regulated by *unc-130*. (**a**) Relationship between the ON/OFF stimulus and calcium dynamics in AIB. During stimulation, excitatory/inhibitory (E/I) balance from ASHs and AIAs could bias inhibition, leading to ON responses (calcium decreases) in AIBs. After stimulation, the ASH OFF response (calcium increase) is a possible trigger for the OFF response (calcium increase) in the AIBs. *unc-130* impaired both ON responses and OFF responses (red line). (**b**) Schematic of the molecules used for optimization and the contribution of *unc-130* (shown in red). *unc-130* is necessary for the glutamatergic signaling pathway because it regulates at least *glr-1*, *nca-2,* and *eat-4* expression in the AIBs to cause defects in the calcium response and turn optimization dynamics.

We have reported that the proneural gene *lin-32* promotes the expression of a wide range of genes in AIBs, including gap junctions^6^. However, *unc-130* seems to play a more limited role in AIB identity. The *unc-130* mutants had reduced expression of *glr-1*, *nca-2*, and *eat-4* in the AIBs but not the electrical synapse component *inx-1* and standard chemical synaptic component SNB-1 in AIB neurons, suggesting that the *unc-130* mutation causes behavioral optimization defects by disrupting selective glutamatergic synapse functions (Fig. 6a).

During intense osmotic stimulation, the AIBs receive excitatory inputs through a direct circuit between the ASHs and AIBs via glutamate and GLR-1 receptors as well as inhibitory inputs mediated by AIAs (Fig. 6a). Considering the AIB suppression that occurs during osmotic stimulation (Fig. 2a), inhibitory input may exceed GLR-1-mediated excitatory input during hyperosmolar stimulation. We could not identify the inhibitory receptors on AIBs in this study (Figs. S4 and S5). In addition to the unanalyzed inhibitory acetylcholine receptors, neuropeptide receptors might be involved in AIA-AIB suppression. The *unc-130* mutation may regulate either inhibitory receptors or genes in the downstream calcium signaling cascade.

The molecular basis of optimization after intense osmotic stimulation is also intriguing. After stimulation, OFF responses (calcium increases) in ASHs can induce glutamate release. GLR-1, a receptor for glutamate on AIBs, induces a change in conductance to open NCA-2, a predicted voltage-gated cation channel or a cation leak channel, in order to promote the intracellular influx of cations. This contributes to the increase in the OFF-calcium concentration response in AIBs. We showed that overexpression of NCA-2a in AIBs of *unc-130* mutants rescued the optimization defect (Fig. 3e), and *unc-130* partially downregulated *nca-2* and *glr-1* (Figs. 3d and 4b). These results imply that excitatory input is reduced and suggest the amplification mechanism.

The vesicular glutamate transporter EAT-4 transports glutamate into synaptic vesicles. Calcium induction promotes the secretion of glutamate-releasing synaptic vesicles that transmit information to downstream inter-/motor neurons, including neck motor neurons. We have shown that the contraction strength of the neck positively correlates with turn behavior^6^. Thus, the AIBs appear to integrate the two excitatory and inhibitory (E/I) pieces of information from the AIAs and ASHs to evaluate the intensity and exposure time of the stimulus and to output avoidance behavior with appropriate intensity and timing.

We demonstrated that *unc-130* mutants show reduced expression of *glr-1* and *nca-2* (Figs. 3c, d, 4a, b), suggesting that insufficient excitatory input from ASHs and voltage-dependent calcium influx occur. Additionally, the reduced expression of EAT-4 may lead to insufficient glutamate loading in the synaptic vesicles, resulting in reduced transmission of information to the downstream neural circuit.

On the other hand, since AIB-specific driven *unc-130* does not rescue behavioral abnormalities (Figs. S3 and S4), showing the importance of an act of *unc-130* in a developmental stage. Often, with the loss of transcription factors during developmental stages, unexpected circuits may form or differentiate into different cells that are similar in lineage. It has also been reported that *unc-130* is required to make a difference between AWA and ASG, chemosensory neurons generated from the ABp(l/r)aapap lineage from which AIB is derived^30^. *unc-130* specifies two glial types that arise from the neighboring lineage (ABp(l/r)aapa)^54^. As for the AIBs that we are focusing on in this study, they express at least *inx-1*, which is selective for AIBs, and show a consistent number, location, and interneuron-like morphology. We consider that they are incomplete differentiated AIBs rather than sister cells of sensory neurons or glial cells.

Now we conclude that *unc-130* is involved in both the function and development of AIBs, and comprehensive discussion summarizing the importance of both sides is more appropriate for the role of *unc-130*. *unc-130* is required for the establishment of some AIB identities (e.g., biphasic response, expression of *nca-2*, *glr-1*, and *eat-4*, and role as a behavioral optimization center), and *unc-130* contributes to behavioral optimization through its role in regulating the expression of a group of genes that are necessary for AIB identities. *unc-130* has the defect in the lineage determination, so the possibility remains that it reflects an unidentified reorganization of neurons in addition to AIB defects. It might explain the synthetic defect in the behavior of *unc-130;lin-32* double mutants.

### Biphasic neural response of AIB neurons

We provide the first evidence for a biphasic neural response of AIBs: calcium decreases during osmotic stimulation followed by a calcium increase after the stimulus (Fig. 2a and Fig. S3a). First, we discuss the neural mechanisms of the calcium decrease in wild-type *C. elegans* during stimulation. Calcium imaging results showed that ASHs and AIAs were excited and that AIBs were inhibited during the stimulus (Fig. 2a, d, and e; Fig. S1a, e and f). Since ASHs and AIAs form synapses and since AIA-AIB connections are likely to be inhibitory (Fig. 2b)^7,32^, we speculate that ASHs may directly excite AIAs during stimulation and that excited AIAs inhibit AIBs (Figs. 2b and 6a), resulting in decreased intracellular calcium concentrations in AIBs (Fig. 6b). It has been implied that excitation of AWC chemosensory neurons might cause calcium decreases in AIBs during stimulus exposure^55,56^, but this has not been stringently verified. This study clearly showed such responses with the indicator IP2.0, which monitors the decrease in intracellular calcium concentration associated with osmotic stimulation received by ASHs.

Next, we considered the neural mechanisms of calcium increases in wild-type *C. elegans* after stimulation. Calcium imaging results showed that ASHs and AIBs, but not AIAs, were excited after the stimulus (Figs. 2a, d, and e; Fig. S3a, e and f). Although removing the CO_2_ stimulus evokes an AIA OFF response (calcium increase)^57^, it is likely due to neural circuits distinct from those involved in response to noxious osmolality. Anatomical analysis showed a direct synaptic connection between the ASHs and AIBs (Fig. 2b)^58^, but the details of ASH-AIB synapses are not well understood. Since ASHs show the biphasic (ON/OFF-increase) response (Fig. 2d)^46^, we speculate that the ASH OFF response (calcium increase) engages the disinhibition of AIB after stimulation^6^.

Physiologically, such a biphasic interneuron response has also been observed in mammals. In rodents, over 60% of suprachiasmatic nucleus neurons may use such “rebound responses” or “postinhibitory rebounds,” and the probability of the response is positively correlated with the duration of hyperpolarization^59^. One of the next questions will be to determine whether the duration of ON-hyperpolarization of AIBs is correlated with the probability of OFF-calcium responses in *C. elegans*, so that similarity with a property of suprachiasmatic nucleus neurons can be clarified. Rebound responses are also observed in striatal neurons and imply the existence of an essential mechanism for fear processing and decision-making^60^. *C. elegans* AIBs share similarities in that they are involved in escape behavior optimization and behavioral choice in response to harmful stimuli. The biphasic neural response with excitatory-inhibitory association seems likely to have been evolutionarily acquired for behavioral diversity. We consider that the biphasic neural response in *C. elegans* may be one of the neural mechanisms of primitive excitation-inhibition association. In addition, rebound firing is also associated with diseases; for example, drug addiction pathogenesis in rodents and thalamic neurons generate unusual rebound firing at the end of inhibition in Parkinson's disease in humans^60,61^. In the future, *C. elegans* AIB neurons may be used as a neuron model for drug addiction pathogenesis and neurodegenerative diseases.

Furthermore, AIB biphasic responses may biologically contribute to temporal control of the initiation of turn behavior. Direct AIB excitation by optogenetics facilitates turning without reversal^6^, suggesting that AIA-AIB inhibition suppresses time-consuming turn behavior under conditions of toxic osmolality to expedite escape.

### Involvement of *FOXD3/4* in avoidance behavior optimization

In this study, we demonstrated, for the first time, that a *FOXD3/4* ortholog, *unc-130*, specifies avoidance behavior patterns using *C. elegans*. *FOXD3* is widely conserved from invertebrates to vertebrates, including humans^10^. *FOXD3* defines the early pluripotency of neural crest stem cells in vertebrates to differentiate into diverse cells, such as neurons and muscles^62–64^. Even in *C. elegans*, *unc-130* has a similarity to *FOXD3/4* with regard to its expression in both neural progenitors and adult muscle cells^30,65,66^. Future *unc-130* studies may lead to universal molecular insights into primary neurogenesis. In addition, whole-human genome analysis has implied that *FOXD4* is a risk factor for suicide and obsessive–compulsive disorder^67^. Such outcomes may be attributable to vulnerability to stress. *unc-130* might be useful for illustrating a prototypical circuit for improved coping behavior under exposure to harmful stimuli.

During development, combinations of transcription factors determine neuron identities. Hobert et al. have comprehensively mapped the combination of transcription factors expressed in all neurons of *C. elegans*^37,68^. They speculate that combinations of homeobox (Hox) transcription factors can code for almost all neural identities^69^; these findings will accelerate the understanding of individual neural characters. In addition, basic helix-loop-helix (bHLH) transcription factors, including proneural genes, act in the initial phase and have a crucial role in neurogenesis^61^. However, forkhead box (Fox) transcription factors were grouped relatively recently in 2000^61^, and their functions in the nervous system are still less known than those of Hox and bHLH molecules.

## Conclusion

Our findings suggest that a *FOXD3/4* ortholog, *unc-130* contributes to behavioral optimization mediated by pre- and postsynaptic function to mediate biphasic neural responses. In summary, reductions in the ON and OFF calcium responses required for integrating this information and producing behavioral outputs result in incorrect behavioral choices in response to stimuli of different intensities in *unc-130* mutants.

## Methods

### Nematodes and maintenance

We cultured *C. elegans* strains using modified standard techniques^70^. NGM agar plates containing 67 mg/ml antibiotic streptomycin and 10 µg/ml nystatin were used. *Escherichia coli* OP50-1 was seeded as food. *unc-130(tm320), acc-1(tm3268), nca-2(tm1305)*, *unc-2(e55)*, and *lin-32(tm2044)* mutants were backcrossed twice with N2. *lgc-46(ok2949)*, *lgc-49(tm6556),* and *goa-1(sa734)* mutants were backcrossed three, four, and five times with N2, respectively. *cca-1(gk30)*, *nca-1(gk9)*, *unc-36(e251)*, *unc-36(ok862)*, and *ccb-2(ok862)* were not backcrossed because of the pilot screening (Fig. S6b). The deletion and point mutation sites are described in Fig. S13. The strain information is summarized in Table S1.

### Plasmid construction

For the own-promoter rescue experiment (Fig. 1h; Figs. S1i–l), pPD_*Punc-130:*:*UNC-130* was constructed by subcloning the sequence from 5854 bp upstream of the ATG to the end of the 3' UTR of the *unc-130* genomic sequence into the pPD95.75 vector instead of *gfp*. For the AIB-specific promoter rescue experiment (Figs. S10 and S11), promoters of *inx-1* or *Pnpr-9*^6^ were transferred into the pPD_*Punc-130:*:*UNC-130* plasmid instead of *Punc-130*. For calcium imaging, we constructed pPD_*Pinx-1::IP2.0* (Fig. 2a; Fig. S3a) or pPD_*Psra-6::GCaMP6s* (Fig. 2d) by subcloning *IP2.0* from pDEST-IP2.0 (codon for *C. elegans*) or both the 2409 bp *sra-6* promoter and *GCaMP6s* sequences from N2 genomic DNA and pGP-CMV-GCaMP6s into the pPD95.75*_Pinx-1*^6^ or pPD95.75 plasmids instead of *gfp*, respectively. For expression analysis of inhibitory acetylcholine receptors (Fig. S4a and b), the 5022 bp *goa-1* promoter, 5354 bp *acc-1* promoter, 5007 bp *lgc-46* promoter, 3485 bp *lgc-49* promoter, 7838 bp *acc-2* promoter, 5777 bp *acc-3* promoter, and 2385 bp *acc-4* promoter sequences upstream of the ATG from N2 genomic DNA were cloned into pPD95.75*_Pinx-1*^6^, respectively.

For expression analysis of *nca-2* (Figs. 3c and d), pPD_*Pnca-2::gfp* was constructed by subcloning a total of 9990 bp containing the *nca-2* promoter and the first exon and intron of the *nca-2a* region using the N2 genome as a template into the pPD95.75 vector^6^. For rescue analysis of *nca-2* (Fig. 3e; Fig. S7), the 5611 bp *nca-2* coding region using the cDNA template into the pPD_P*inx-1*::*gfp* instead of *gfp*. For rescue analysis of *eat-4* (Fig. S13), the 2218 bp *eat-4* coding region using the cDNA template into the pPD_P*inx-1*::*gfp* instead of *gfp*. For observation of synaptic localization (Figs. 4e–g), we constructed pPD_*Pinx-1::snb-1::mCherry* by subcloning the 327 bp snb-1 coding region using the cDNA template except for the stop codon into the pPD_*Pinx-1::mCherry* vector^6^. We constructed all plasmids using In-Fusion HD Cloning Plus (Takara Bio USA, 638909). pPD95.75 was a gift from Dr. Andrew Fire. *lin-44p::gfp* was a gift from Dr. Yuichi Iino. pPD_*gcy-28dp::ced-3*(p15), pPD_*gcy-28dp::ced-3*(p17), pPD_P*ins-1(short)::mCherry* (Fig. 2c; Fig. S3b) and pFX_*Pgcy-28.d::GCaMP6s* (Fig. 2e; Fig. S3c and d) were gifts from Dr. Yuji Suehiro. pcDNA3.1/hChR2(H134R)-mCherry (Plasmid #20938), G-CaMP3 (Plasmid #22692), and pGP-CMV-GCaMP6s (Plasmid #40753) were obtained from Addgene (www.addgene.org).

### Transgenic lines and strains

For all rescue experiments, we created three or more independent transgenic lines. For *unc-130* rescue experiments, to generate *tm320;tmEx5292* (Fig. 1g; Fig. S1i–l)*, tm320;jskEx0002*, *tm320;jskEx0003, tm320;jskEx0024, tm320;jskEx0028, tm320;jskEx0029, tm320;jskEx0011, tm320;jskEx0013* and *tm320;jskEx0014* transgenic animals, pPD_*Punc-130*::*UNC-130::3' UTR* (2 ng/μl), pPD_*Pinx-1*::*UNC-130::3' UTR* (2 ng/μl or 0.2 ng/μl) or pPD_*Pnpr-9*::*UNC-130::3' UTR* (2 ng/μl), pPD95.75_*Pinx-1* (20 ng/μl), and pBluescript KS( +)T1 (140 ng/μl) were coinjected with *lin-44p::gfp* (20 ng/μl) as an injection marker into *tm320* mutants, respectively. For calcium imaging, to generate *tmEx5274* transgenic animals, pPD_*Pinx-1::IP2.0* (20 ng/μl) and pBluescript KS( +)T1 (160 ng/μl) were coinjected with *lin-44p::gfp* (20 ng/μl) into N2 animals. For AIA ablation experiments, to generate *tmEx3494* transgenic animals, pPD_*odr-2p*::*ced-3(p15)* (80 ng/μl), pPD_*ser-2p*::*ced-3(p17)* (80 ng/μl), and pFX_*DsRedxT-gpa-2(aa1* + *int.)* (20 ng/μl) were coinjected with *lin-44p::gfp* (20 ng/μl) into N2 animals. To generate *tmEx5137* transgenic animals for ASH calcium imaging, pPD_*Psra-6::GCaMP6s* (180 ng/μl) was coinjected with *lin-44p::gfp* (20 ng/μl) into N2 animals. To generate the *tmEx5293* transgenic animals, pFX_*Pgcy-28.d::GCaMP6s* (50 ng/μl), pPD_*Pins-1*(short)::*mCherry* (30 ng/μl), and pBluescript KS( +)T1 (100 ng/μl) were coinjected with *lin-44p::gfp* (20 ng/μl) into N2 animals. For expression analysis of inhibitory acetylcholine receptors, *acc-1, acc-2, acc-3*, and *acc-4*, to generate *tmEx5408, tmEx5409, tmEx5411*, and *tmEx5412* transgenic animals, pPD_*Pacc-1*::*gfp* (100 ng/μl) or pPD_*Pacc-2*::*gfp* (100 ng/μl), pPD_*Pacc-3*::*gfp* (100 ng/μl) or pPD_*Pacc-4*::*gfp* (100 ng/μl), and pPD_*Pinx-1::mCherry* (80 ng/μl) were coinjected with *lin-44p::gfp* (20 ng/μl) into N2 animals. For *lgc-46* and *lgc-49,* to generate *tmEx5454* and *tmEx5449* transgenic animals, pPD_*Plgc-46*::*gfp* (100 ng/μl) or pPD_*Plgc-49*::*gfp* (100 ng/μl), pPD_*Pinx-1::mCherry* (40 ng/μl), and pBluescript KS( +)T1 (40 ng/μl) were coinjected with *lin-44p::gfp* (20 ng/μl) into N2 animals. To generate *tmEx5614* transgenic animals, pPD_*Pgoa-1*::*gfp* (100 ng/μl), pPD_*Pinx-1::mCherry* (20 ng/μl) and pBluescript KS( +)T1 (60 ng/μl) were coinjected with *lin-44p::gfp* (20 ng/μl) into N2 animals. For expression analysis of the hypothetical voltage-dependent calcium channels and *nca-2,* to generate *tmEx5615* and transgenic animals, pPD_*Pnca-2*::*gfp* (100 ng/μl) and pPD_*Pinx-1::mCherry* (40 ng/μl) were coinjected with *lin-44p::gfp* (20 ng/μl) and pBluescript KS( +)T1 (40 ng/μl) into N2 animals. For AIB-specific rescue, to generate *tm1305; tmEx5629, tm1305;jskEx0017* and *tm1305;jskEx0018* transgenic animals, pPD_*Pinx-1*::*NCA-2a* (20 ng/μl) and pPD_*Pinx-1::gfp* (20 ng/μl) were coinjected with *lin-44p::gfp* (20 ng/μl) and pBluescript KS( +)T1 (140 ng/μl) into *nca-2(tm1305)* animals. To generate *tm1305;jskEx0019*, *tm1305;jskEx0020 and tm1305;jskEx0021* transgenic animals, pPD_*Pinx-1*::*EAT-4* (20 ng/μl) and pPD_*Pinx-1::gfp* (20 ng/μl) were coinjected with *lin-44p::gfp* (20 ng/μl) and pBluescript KS( +)T1 (140 ng/μl) into *unc-130(tm320)* animals. For precise synaptic localization, to generate *tmEx5377* transgenic animals, pPD_*Pinx-1*::*SNB-1::mCherry* (80 ng/μl) and pPD_*Pinx-1::gfp* (20 ng/μl) were coinjected with *unc-122p::mCherry* (100 ng/μl) into N2 animals. The strains *tmIs825[Psra-6::ChR2(H134R)::mCherry* + *Pges-1::EGFP]*), *tmEx4532[Pnpr-4::G-CaMP6s* + *Pnmr-1::mCherry* + *Pin-44::gfp]*, *tmEx4456[Pinx-1::ChR2(H134R)::mCherry* + *Pinx-1::gfp* + *Plin-44::gfp]*, *rhIs4[glr-1::GFP* + *dpy(* +*)]; tmEx3532[DsRedxT-odr-2(aa1)* + *pBluescript KS* + *T1]* (Fig. 4a and b), *otIs292[eat-4::mCherry* + *rol-6]; tmEx3958[Pinx-1::gfp* + *Plin-44::gfp* + *pBluescript]* (Fig. 4c and d), and *tmIs1260[Pinx-1::gfp* + *Punc-122::mCherry]* were generated in our previous study^6^. *tmIs825*, *tmIs1260*, and *rhIs4* were backcrossed twice, and *otIs292* was backcrossed once with N2. Supplemental Table 1 summarizes the strain names, genotypes, injected plasmid concentrations, injected recipients, number of outcross with N2, and methods of crossing with mutants of the alleles used in the paper.

### Drop test

We performed a drop test as we have previously described^6^. In this study, we used 1–6 M sorbitol dissolved in S basal^6^. “n = x” in the figure legends indicates the number of plates analyzed. Responses were classified as omega turns, long reversals, or short reversals, as previously described^6^. Each score was calculated as the average percentage for 10 ± 3 animals. For a rescue experiment, we selected *Pinx-1::gfp* marker-positive animals as AIB-rescued animals since extrachromosomal transgenic animals exhibited a mosaic expression pattern. The experimenter was blinded to the nematode strains during the experiment.

### Harsh tap assay with a platinum wire

We tapped the nematodes on the tip of their noses with a platinum wire (diameter: 0.25 mm) on NGM plates, respectively. The platinum wire has a smooth polished surface to avoid damaging the nematode. “n = x” in the figure legends indicates the number of plates analyzed. Responses were classified as omega turns and others, as previously described^6^. Each score was calculated as the average percentage for 10 animals. The experimenter was blinded to the nematode strains during the experiment.

### Channelrhodopsin 2-induced avoidance assay

We performed a ChR2(H134R)-induced avoidance assay as previously described^6,71^. The animals were individually irradiated with 100% blue light (approximately 2.47 μW/cm^2^) using a CFP filter (365 nm) at their heads for 2 s. Each score was calculated for 10 ± 3 animals. We performed the experiments on at least three different days and calculated the average percentage. “n = x” in the figure legends indicates the number of plates analyzed. The experimenter was blinded to the nematode strains and to whether all-trans retinoic acid (ATR) was added or not.

### Calcium imaging of neurons

As previously described, we performed calcium imaging using an olfactory chip^6,13^. We used 2 M sorbitol dissolved in S basal as a stimulus. All optical recordings of neurons were performed on an IX71 microscope with a 40X immersion objective (Olympus Optical) and an ORCA-Flash2.8 CMOS camera (Hamamatsu Photonics) and analyzed with MetaMorph software (Molecular Devices). We captured time stacks of the fluorescence images at one frame per second. The images were analyzed as previously described^18^. We calculated the percent change in the fluorescence intensity relative to the average intensity during the 5 s before stimulation. In the IP2.0 analysis, the same baseline fluorescence was seen in both wild-type animals and *unc-130* mutants, we shifted them to 0 for subtraction and normalization as corrected baselines. In the statistical analysis of AIA, we compared the maximum ΔF/F values from the 5 s before stimulation to 100 s after stimulation. We performed image tracking using a custom ImageJ (NIH, https://imagej.nih.gov/ij/) plugin. We drew a rectangular region of interest (ROI) surrounding the cell body, and the ROI was shifted according to the new position of the center for every frame.

### Microscopy

Nematodes were immobilized with M9 buffer containing 50 mM sodium azide on a 5% agarose pad containing 10 mM sodium azide. We obtained fluorescence images (Figs. 3c and 4a; Figs. S4a, b, S5b, and S8a) using a BX51 microscope equipped with a DP30BW CCD camera (Olympus Optical). Confocal microscopic images were captured with Zeiss LSM710 confocal microscopes with either 40X oil immersion objectives for single-plane projections (Fig. 4c) or Z-stacks spanning the focal depths (1 μm/step) of the AIB neurons (Figs. 4e and 5a) using ZEN 2011 software (Zeiss, https://www.zeiss.co.jp/microscopy/downloads/zen.html). We drew an ROI surrounding the cell body (Figs. 3d and 4d; Fig. S2c) or neurite (Figs. 4g and 5b) and measured the total fluorescence intensity using a custom ImageJ (NIH, https://imagej.nih. gov/ij/) plugin. We performed ratiometric analysis for an accurate discussion of expression levels in extrachromosomal arrays, comparing marker fluorescence to *nca-2* (GFP) (Fig. 3d), SNB-1(mCherry) (Fig. 4e), and *goa-1* (GFP) (Fig. S5c) fluorescence by drawing the same ROI position. We calculated the synapse dimensions (Fig. 4f) using a custom ImageJ plugin.

### Quantification and statistical analysis

We performed statistical analyses using GraphPad Prism 6 (GraphPad Software). Pairwise comparisons of omega turn frequencies within two groups were carried out via Student's *t*-test, Mann–Whitney test, or Fisher’s exact test according to the results of the Shapiro–Wilk normality test. We used one-way ANOVA followed by Tukey’s post hoc test or the Kruskal–Wallis test with Dunn's multiple comparisons test as a post hoc test according to the results of the Shapiro–Wilk normality test for multiple groups. We produced bar graphs with the mean ± SEM values from three or more independent experiments. In both cases, “n” is the number of plates (cohorts) of 10 ± 3 animals each. In the bar graphs without error bars (Fig. 4b), “n” is the number of individuals (animals). We have provided the statistical information and the total number of experiments, animals, or cells analyzed per experiment in the figure legends.

## Supplementary Information

Supplementary Information 1.

Supplementary Information 2.

## References

[1] Baliki MN, Apkarian AV (2015). Nociception, pain, negative moods, and behavior selection. Neuron.

[2] Pirri JK, Alkema MJ (2012). The neuroethology of *C. elegans* escape. Curr. Opin. Neurobiol..

[3] Bargmann CI, Kaplan JM (1998). Signal transduction in the Caenorhabditis elegans nervous system. Ann. Rev. Neurosci..

[4] Eaton RC, Lee RK, Foreman MB (2001). The Mauthner cell and other identified neurons of the brainstem escape network of fish. Prog. Neurobiol..

[5] Hale ME, Katz HR, Peek MY, Fremont RT (2016). Neural circuits that drive startle behavior, with a focus on the Mauthner cells and spiral fiber neurons of fishes. J. Neurogenet..

[6] Hori S, Oda S, Suehiro Y, Iino Y, Mitani S (2018). OFF-responses of interneurons optimize avoidance behaviors depending on stimulus strength via electrical synapses. PLoS Genet..

[7] White JG, Southgate E, Thomson JN, Brenner S (1986). The structure of the nervous system of the nematode Caenorhabditis elegans. Philos. Trans. R Soc. Lond. B Biol. Sci..

[8] Liu H, Kim J, Shlizerman E (2018). Functional connectomics from neural dynamics: probabilistic graphical models for neuronal network of Caenorhabditis elegans. Philos. Trans. R Soc. Lond. B Biol. Sci..

[9] Shaye DD, Greenwald I (2011). OrthoList: a compendium of *C. elegans* genes with human orthologs. PLoS ONE.

[10] Kim W, Underwood RS, Greenwald I, Shaye DD (2018). OrthoList 2: a new comparative genomic analysis of human and Caenorhabditis elegans Genes. Genetics.

[11] Pokala N, Liu Q, Gordus A, Bargmann CI (2014). Inducible and titratable silencing of Caenorhabditis elegans neurons in vivo with histamine-gated chloride channels. Proc. Natl. Acad. Sci. U S A.

[12] Hammarlund M, Hobert O, Miller DM, Sestan N (2018). The CeNGEN project: the complete gene expression map of an entire nervous system. Neuron.

[13] Chronis N, Zimmer M, Bargmann CI (2007). Microfluidics for in vivo imaging of neuronal and behavioral activity in Caenorhabditis elegans. Nat. Methods.

[14] Baker NE, Brown NL (2018). All in the family: proneural bHLH genes and neuronal diversity. Development.

[15] Mitani S, Du H, Hall DH, Driscoll M, Chalfie M (1993). Combinatorial control of touch receptor neuron expression in Caenorhabditis elegans. Development.

[16] Deneris ES, Hobert O (2014). Maintenance of postmitotic neuronal cell identity. Nat. Neurosci..

[17] Powell LM, Jarman AP (2008). Context dependence of proneural bHLH proteins. Curr. Opin. Genet. Dev..

[18] Wang W, Xu ZJ, Wu YQ, Qin LW, Li ZY, Wu ZX (2015). Off-response in ASH neurons evoked by CuSO4 requires the TRP channel OSM-9 in Caenorhabditis elegans. Biochem. Biophys. Res. Commun..

[19] Topalidou I, Chen PA, Cooper K, Watanabe S, Jorgensen EM, Ailion M (2017). The NCA-1 and NCA-2 Ion channels function downstream of Gq and Rho to regulate locomotion in Caenorhabditis elegans. Genetics.

[20] Hart AC, Sims S, Kaplan JM (1995). Synaptic code for sensory modalities revealed by *C. elegans* GLR-1 glutamate receptor. Nature.

[21] Maricq AV, Peckol E, Driscoll M, Bargmann CI (1995). Mechanosensory signalling in *C. elegans* mediated by the GLR-1 glutamate receptor. Nature.

[22] Lee D, Jung S, Ryu J, Ahnn J, Ha I (2008). Human vesicular glutamate transporters functionally complement EAT-4 in *C. elegans*. Mol. Cells.

[23] Nonet ML, Saifee O, Zhao H, Rand JB, Wei L (1998). Synaptic transmission deficits in Caenorhabditis elegans synaptobrevin mutants. J. Neurosci..

[24] Hata Y, Slaughter CA, Sudhof TC (1993). Synaptic vesicle fusion complex contains unc-18 homologue bound to syntaxin. Nature.

[25] Sandoval GM, Duerr JS, Hodgkin J, Rand JB, Ruvkun G (2006). A genetic interaction between the vesicular acetylcholine transporter VAChT/UNC-17 and synaptobrevin/SNB-1 in *C. elegans*. Nat. Neurosci..

[26] Gray JM, Hill JJ, Bargmann CI (2005). A circuit for navigation in Caenorhabditis elegans. Proc. Natl. Acad. Sci. U S A.

[27] Chen G, Ishan M, Yang J, Kishigami S, Fukuda T, Scott G, Ray MK, Sun C, Chen SY, Komatsu Y (2017). Specific and spatial labeling of P0-Cre versus Wnt1-Cre in cranial neural crest in early mouse embryos. Genesis.

[28] Backonja M-M, Schmidt RF, Willis WD (2007). Hypoesthesia, assessment. Encyclopedia of Pain.

[29] Nagel G, Brauner M, Liewald JF, Adeishvili N, Bamberg E, Gottschalk A (2005). Light activation of channelrhodopsin-2 in excitable cells of Caenorhabditis elegans triggers rapid behavioral responses. Curr. Biol..

[30] Sarafi-Reinach TR, Sengupta P (2000). The forkhead domain gene unc-130 generates chemosensory neuron diversity in *C. elegans*. Genes Dev..

[31] Hara-Kuge S, Nishihara T, Matsuda T, Kitazono T, Teramoto T, Nagai T, Ishihara T (2018). An improved inverse-type Ca^2+^ indicator can detect putative neuronal inhibition in Caenorhabditis elegans by increasing signal intensity upon Ca^2+^ decrease. PLoS ONE.

[32] Wakabayashi T, Kitagawa I, Shingai R (2004). Neurons regulating the duration of forward locomotion in Caenorhabditis elegans. Neurosci. Res..

[33] Chelur DS, Chalfie M (2007). Targeted cell killing by reconstituted caspases. Proc. Natl. Acad. Sci. U S A.

[34] Chen TW, Wardill TJ, Sun Y, Pulver SR, Renninger SL, Baohan A, Schreiter ER, Kerr RA, Orger MB, Jayaraman V (2013). Ultrasensitive fluorescent proteins for imaging neuronal activity. Nature.

[35] Ding J, Luo AF, Hu L, Wang D, Shao F (2014). Structural basis of the ultrasensitive calcium indicator GCaMP6. Sci. China Life Sci..

[36] Dobosiewicz M, Liu Q, Bargmann CI (2019). Reliability of an interneuron response depends on an integrated sensory state. Elife.

[37] Hobert O (2016). A map of terminal regulators of neuronal identity in Caenorhabditis elegans. Wiley Interdiscip. Rev. Dev. Biol..

[38] Putrenko I, Zakikhani M, Dent JA (2005). A family of acetylcholine-gated chloride channel subunits in Caenorhabditis elegans. J. Biol. Chem..

[39] Takayanagi-Kiya S, Zhou K, Jin Y (2016). Release-dependent feedback inhibition by a presynaptically localized ligand-gated anion channel. Elife.

[40] Lochrie MA, Mendel JE, Sternberg PW, Simon MI (1991). Homologous and unique G protein alpha subunits in the nematode Caenorhabditis elegans. Cell Regul..

[41] Park YS, Lee YS, Cho NJ, Kaang BK (2000). Alternative splicing of gar-1, a Caenorhabditis elegans G-protein-linked acetylcholine receptor gene. Biochem. Biophys. Res. Commun..

[42] Lee YS, Park YS, Nam S, Suh SJ, Lee J, Kaang BK, Cho NJ (2000). Characterization of GAR-2, a novel G protein-linked acetylcholine receptor from Caenorhabditis elegans. J. Neurochem..

[43] Park Y-S, Kim S, Shin Y, Choi B, Cho NJ (2003). Alternative splicing of the muscarinic acetylcholine receptor GAR-3 in Caenorhabditis elegans. Biochem. Biophys. Res. Commun..

[44] Steger KA, Avery L (2004). The GAR-3 muscarinic receptor cooperates with calcium signals to regulate muscle contraction in the Caenorhabditis elegans pharynx. Genetics.

[45] Kuhara A, Ohnishi N, Shimowada T, Mori I (2011). Neural coding in a single sensory neuron controlling opposite seeking behaviours in Caenorhabditis elegans. Nat. Commun..

[46] Hobert O: The neuronal genome of Caenorhabditis elegans. *WormBook* 2013:1–106. 10.1895/wormbook.1.161.1 PMC4781646 24081909

[47] Laine V, Frokjaer-Jensen C, Couchoux H, Jospin M (2011). The alpha1 subunit EGL-19, the alpha2/delta subunit UNC-36, and the beta subunit CCB-1 underlie voltage-dependent calcium currents in Caenorhabditis elegans striated muscle. J. Biol. Chem..

[48] Schafer WR, Kenyon CJ (1995). A calcium-channel homologue required for adaptation to dopamine and serotonin in Caenorhabditis elegans. Nature.

[49] Shtonda B, Avery L (2005). CCA-1, EGL-19 and EXP-2 currents shape action potentials in the Caenorhabditis elegans pharynx. J. Exp. Biol..

[50] Zou W, Fu J, Zhang H, Du K, Huang W, Yu J, Li S, Fan Y, Baylis HA, Gao S (2018). Decoding the intensity of sensory input by two glutamate receptors in one *C. elegans* interneuron. Nat. Commun..

[51] Pereira L, Kratsios P, Serrano-Saiz E, Sheftel H, Mayo AE, Hall DH, White JG, LeBoeuf B, Garcia LR, Alon U (2015). A cellular and regulatory map of the cholinergic nervous system of *C. elegans*. Elife.

[52] Mahoney TR, Liu Q, Itoh T, Luo S, Hadwiger G, Vincent R, Wang ZW, Fukuda M, Nonet ML (2006). Regulation of synaptic transmission by RAB-3 and RAB-27 in Caenorhabditis elegans. Mol. Biol. Cell.

[53] Kersey RK, Brodigan TM, Fukushige T, Krause MW (2016). Regulation of UNC-130/FOXD-mediated mesodermal patterning in *C. elegans*. Dev. Biol..

[54] Mizeracka K (2021). Lineage-specific control of convergent differentiation by a Forkhead repressor. Development.

[55] Chalasani SH, Chronis N, Tsunozaki M, Gray JM, Ramot D, Goodman MB, Bargmann CI (2007). Dissecting a circuit for olfactory behaviour in Caenorhabditis elegans. Nature.

[56] Wang L, Sato H, Satoh Y, Tomioka M, Kunitomo H, Iino Y (2017). A gustatory neural circuit of Caenorhabditis elegans generates memory-dependent behaviors in Na(+) chemotaxis. J. Neurosci..

[57] Fenk LA, de Bono M (2015). Environmental CO2 inhibits Caenorhabditis elegans egg-laying by modulating olfactory neurons and evokes widespread changes in neural activity. Proc. Natl. Acad. Sci. U S A.

[58] Metaxakis A, Petratou D, Tavernarakis N (2018). Multimodal sensory processing in Caenorhabditis elegans. Open Biol..

[59] Tremere LA, Pinaud R, Irwin RP, Allen CN (2008). Postinhibitory rebound spikes are modulated by the history of membrane hyperpolarization in the SCN. Eur. J. Neurosci..

[60] Ibanez-Sandoval O, Tecuapetla F, Unal B, Shah F, Koos T, Tepper JM (2010). Electrophysiological and morphological characteristics and synaptic connectivity of tyrosine hydroxylase-expressing neurons in adult mouse striatum. J. Neurosci..

[61] Kaestner KH, Knochel W, Martinez DE (2000). Unified nomenclature for the winged helix/forkhead transcription factors. Genes Dev..

[62] Guo Y, Costa R, Ramsey H, Starnes T, Vance G, Robertson K, Kelley M, Reinbold R, Scholer H, Hromas R (2002). The embryonic stem cell transcription factors Oct-4 and FoxD3 interact to regulate endodermal-specific promoter expression. Proc. Natl. Acad. Sci. U S A.

[63] Sherman JH, Karpinski BA, Fralish MS, Cappuzzo JM, Dhindsa DS, Thal AG, Moody SA, LaMantia AS, Maynard TM (2017). Foxd4 is essential for establishing neural cell fate and for neuronal differentiation. Genesis.

[64] Wang LJ, Wang WL, Gao H, Bai YZ, Zhang SC (2018). FOXD3/FOXD4 is required for the development of hindgut in the rat model of anorectal malformation. Exp. Biol. Med. (Maywood).

[65] Nash B, Colavita A, Zheng H, Roy PJ, Culotti JG (2000). The forkhead transcription factor UNC-130 is required for the graded spatial expression of the UNC-129 TGF-beta guidance factor in *C. elegans*. Genes Dev..

[66] Murray JI, Boyle TJ, Preston E, Vafeados D, Mericle B, Weisdepp P, Zhao Z, Bao Z, Boeck M, Waterston RH (2012). Multidimensional regulation of gene expression in the *C. elegans* embryo. Genome Res..

[67] Minoretti P, Arra M, Emanuele E, Olivieri V, Aldeghi A, Politi P, Martinelli V, Pesenti S, Falcone C (2007). A W148R mutation in the human FOXD4 gene segregating with dilated cardiomyopathy, obsessive-compulsive disorder, and suicidality. Int. J. Mol. Med..

[68] Hobert O (2016). Terminal selectors of neuronal identity. Curr. Top. Dev. Biol..

[69] Reilly MB, Cros C, Varol E, Yemini E, Hobert O (2020). Unique homeobox codes delineate all the neuron classes of *C. elegans*. Nature.

[70] Brenner S (1974). The genetics of Caenorhabditis elegans. Genetics.

[71] Guo ZV, Hart AC, Ramanathan S (2009). Optical interrogation of neural circuits in Caenorhabditis elegans. Nat. Methods.

