# Supplementary material for: The transcription factor unc-130/FOXD3/4 contributes to the biphasic calcium response required to optimize avoidance behavior
Source: Sci Rep. 2022 Feb 3;12:1907. doi: 10.1038/s41598-022-05942-0 (PMC8814005; doi:10.1038/s41598-022-05942-0)
Supplement: Supplementary file 2 — Supplementary Information 2. [file 41598_2022_5942_MOESM2_ESM.pdf]

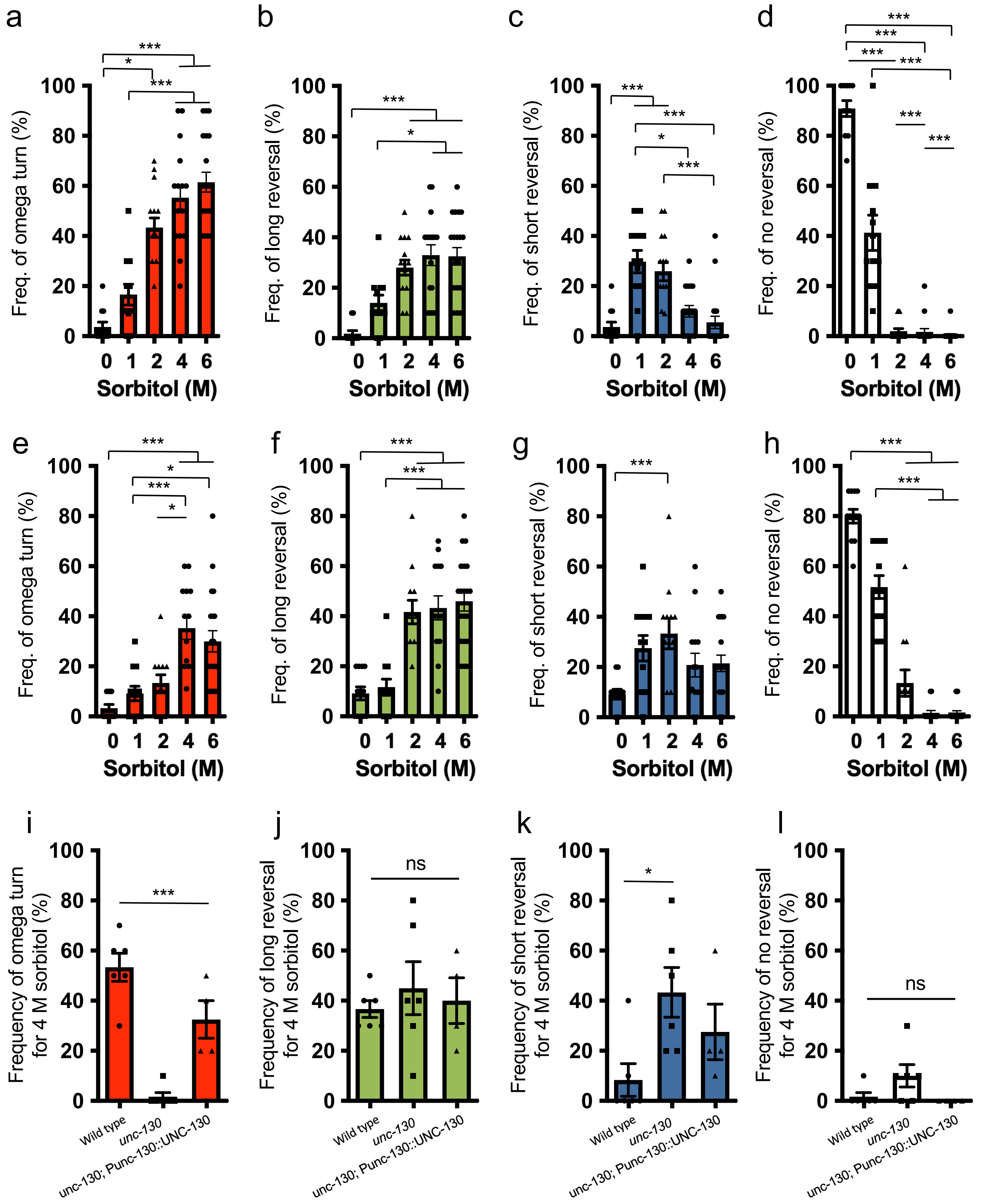

Supplementary Fig. 1 Scatterplots of each behavioral frequency across all replicates (mean ± SEM indicated). a-d Omega turn, long reversal, short reversal and no reversal rates related for Fig. 1b, respectively. **e-h** Omega turn, long reversal, short reversal and no reversal rates related for Fig. 1c, respectively. **i-l** Omega turn, long reversal, short reversal and no reversal rates related for Fig. 1h, respectively. ***indicates $P < 0.001$, **indicates $P < 0.01$, *indicates $P < 0.05$, ns indicates $P > 0.05$.

Supplementary Figure 1 S Hori and S Mitani

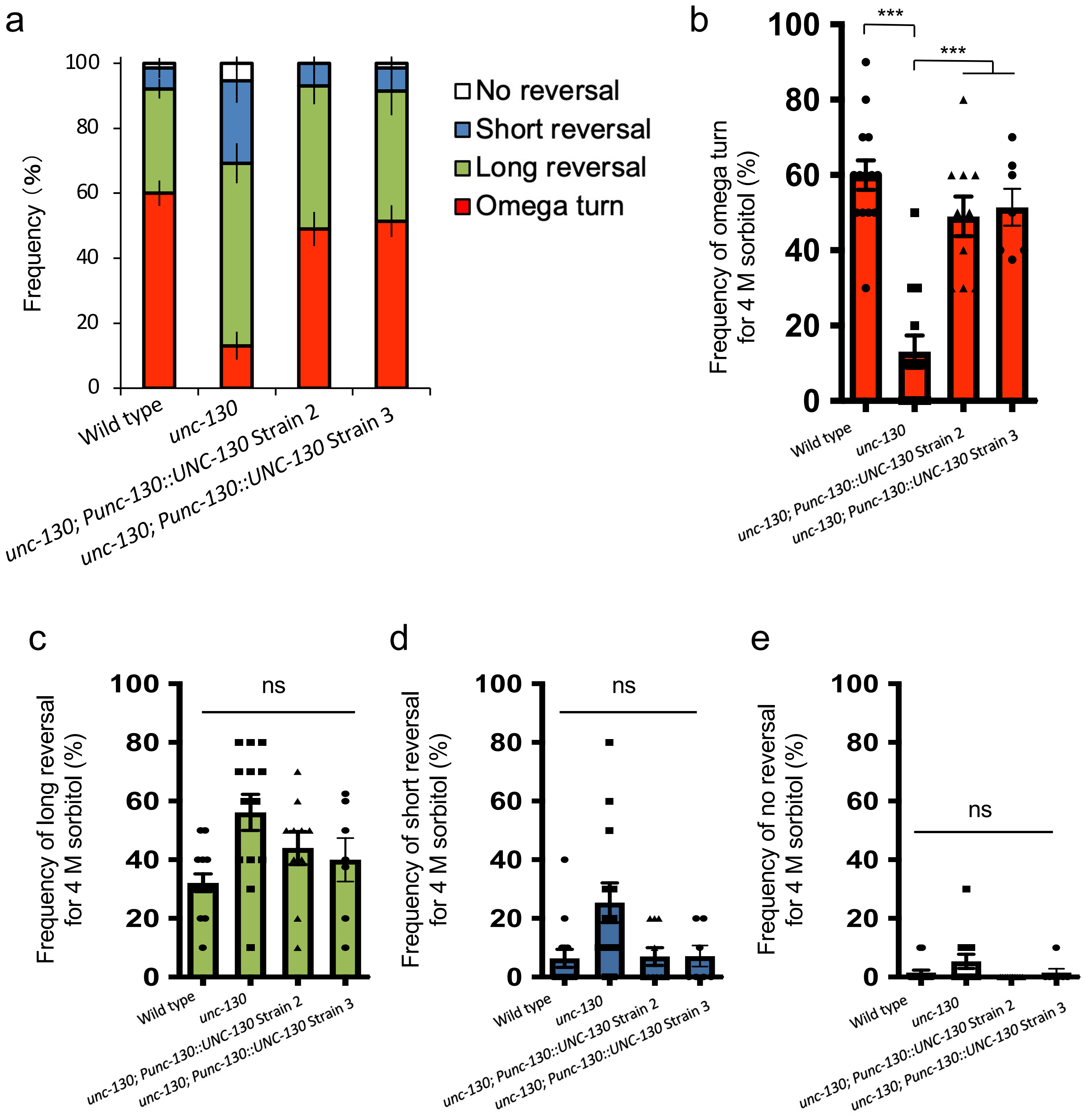

Supplementary Fig. 2 Results of two additional lines for P*unc-130*::UNC-130 rescue, related for Fig. 1h. **a**. Both a strain 2 (*tm320;jskEx0002*) and a strain 3 (*tm320;jskEx0003*) rescued *unc-130* behavioral phenotypes (n = 14, 13, 10, 7)**. b-e** Scatterplots of each behavioral frequency across all replicates (mean ± SEM indicated). Omega turn, long reversal, short reversal and no reversal rates, respectively. ***indicates P < 0.001 and ns indicates P > 0.05 (one-way ANOVA followed by Tukey's post hoc test).

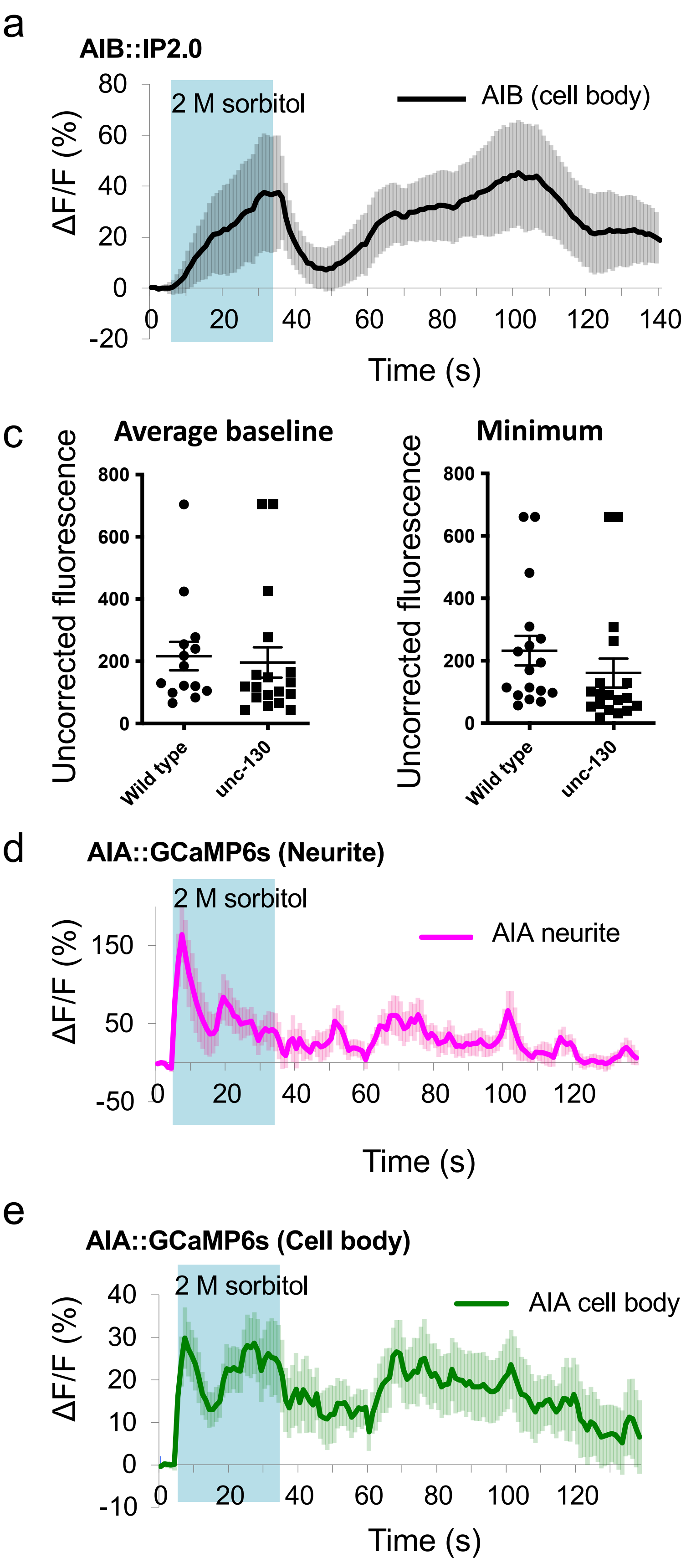

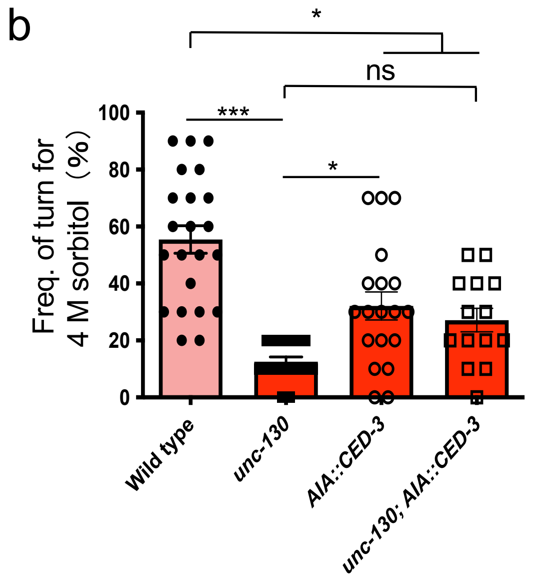

Supplementary Fig. 3 Calcium concentration changes in AIA negatively correlated with the AIB response. a Calcium response of AIBs as determined using the calcium indicator IP2.0 (n = 16). The calcium concentration gradually decreased during stimulation (5-35 sec). An increase in calcium concentration occurred for approximately 20 sec (40-60 sec) after stimulus termination. Then, the calcium concentration returned to a steady state. **b** Scatterplots of all replicates (mean ± SEM indicated), related for Fig. 2c. **c** The baseline fluorescence values. The same baseline fluorescence was seen in both wild-type animals and unc-130 mutants. **d** The minimum uncorrected fluorescence values. There is no significant difference between wild-type animals and *unc-130* mutants in baseline and minimum uncorrected fluorescence values (n = 14, 18, t-test, p = 0.770 or 0.289, respectively). **e** Calcium responses of neurites in AIA interneurons (n = 21). In the early phase of stimulation, an ON response (transient calcium increase) occurred. **f** Calcium responses in the cell bodies of AIA interneurons (n = 20). During stimulation, a sustained increase in calcium concentration occurred. After stimulation, the OFF response (calcium reduction) occurred for approximately 20 sec (40-60 sec in Fig. S1e). After 60 sec after stimulation, the calcium concentration became high again. The error bars in these figures represent the ± SEM values.

Supplementary Figure 3

S Hori and S Mitani

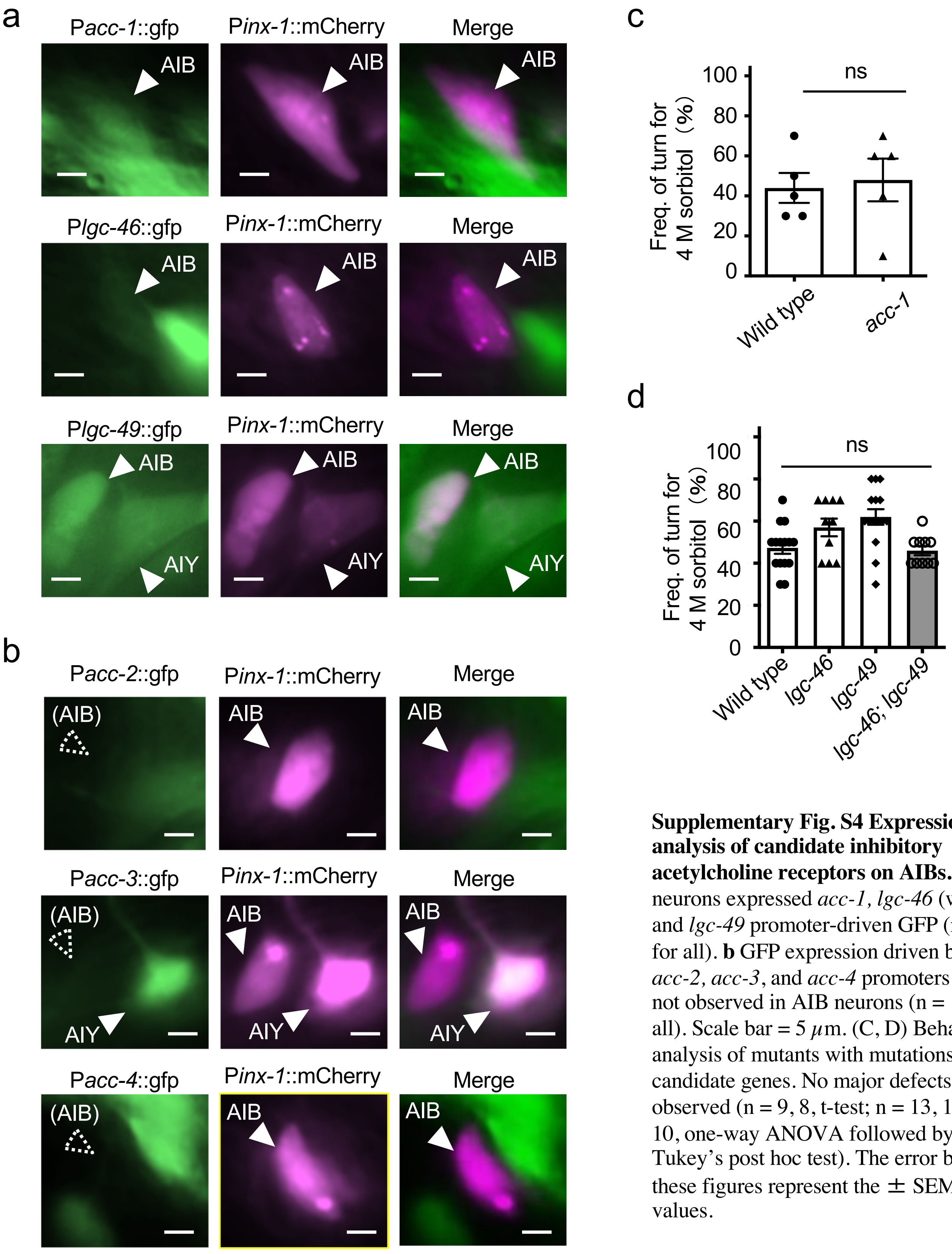

Supplementary Fig. S4 Expression analysis of candidate inhibitory acetylcholine receptors on AIBs. a AIB neurons expressed *acc-1, lgc-46* (weak), and *lgc-49* promoter-driven GFP (n > 3 for all). **b** GFP expression driven by the *acc-2*, *acc-3*, and *acc-4* promoters was not observed in AIB neurons (n = 10 for all). Scale bar = 5 μm. (C, D) Behavioral analysis of mutants with mutations in candidate genes. No major defects were observed (n = 9, 8, t-test; n = 13, 10, 10, 10, one-way ANOVA followed by Tukey's post hoc test). The error bars in these figures represent the ± SEM values.

Supplementary Figure 4

S Hori and S Mitani

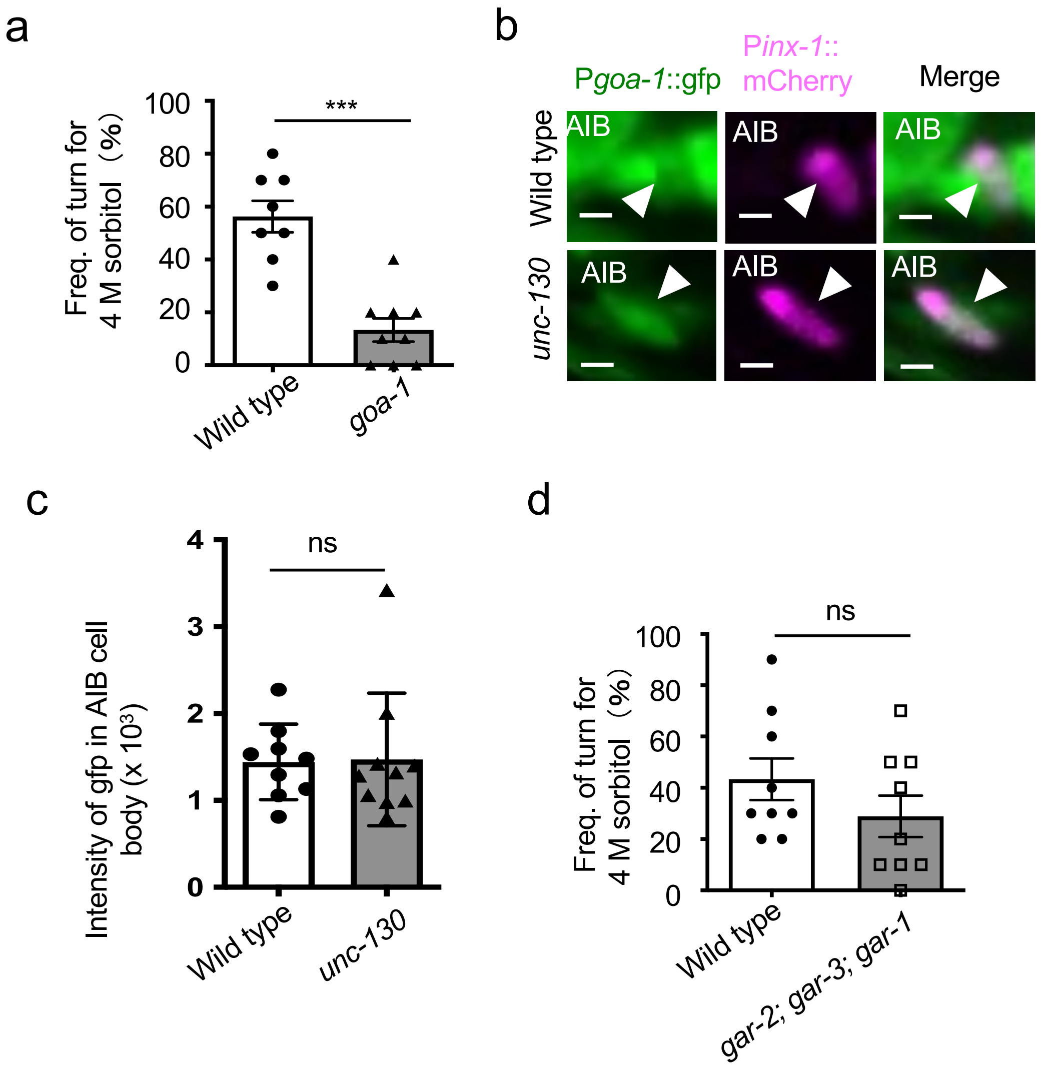

Supplementary Fig. S5 Examination of inhibitory acetylcholine receptors for optimization regulated by *unc-130*. a The *goa-1* deletion mutants showed lower turn frequencies than the wild-type animals. ***indicates $P < 0.001$ (n = 9, 8, t-test). **b, c** AIBs expressed *goa-1* promoter-driven GFP, but the expression was independent of *unc-130* regulation (P = 0.922, N= 9, 10, t-test). Scale bar = 5 μm. **d** The triple mutants of *gar-1*, *gar-2* and *gar-3* showed no significant differences in turn frequency. P = 0.226 (n = 9, 8, t-test). The error bars in these figures represent the ± SEM values.

Supplementary Figure 5

S Hori and S Mitani

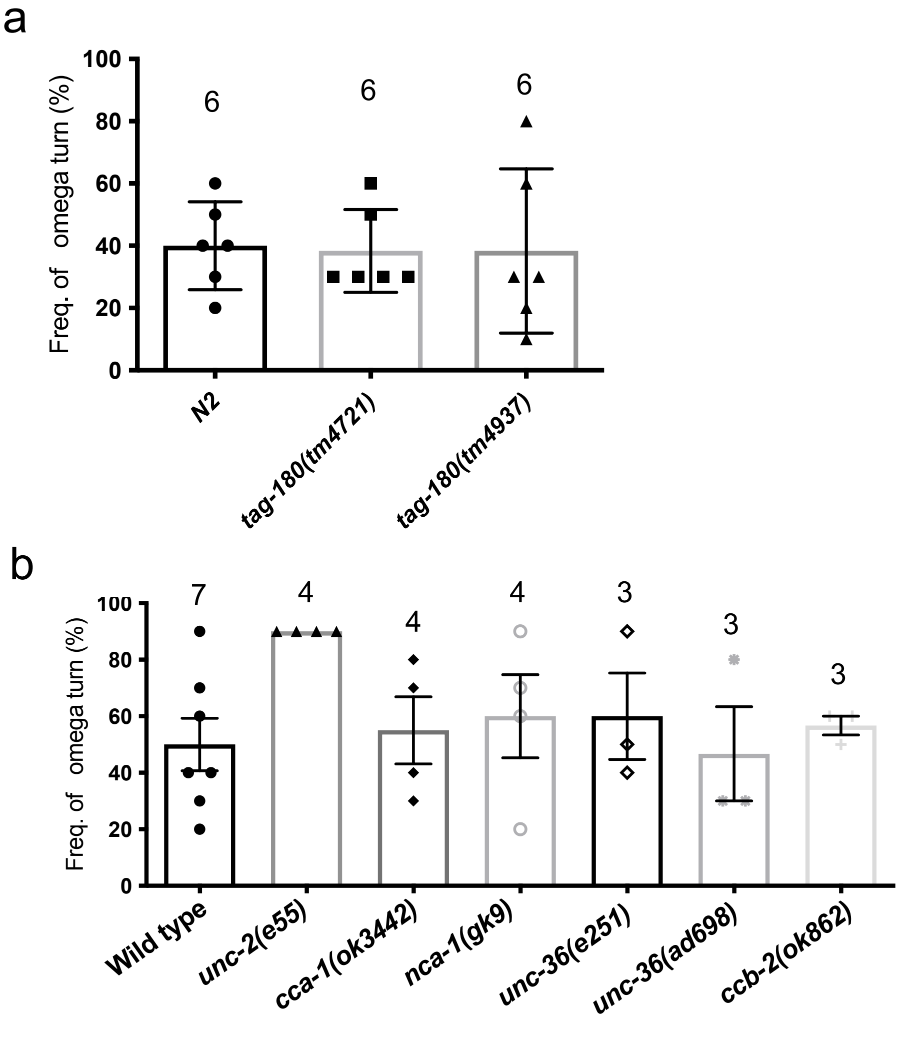

Supplementary Fig. S6 Hypothetical voltage-gated calcium channel subunits do not affect turn frequency. a Behavioral screening of two *tag-180* mutants based on turn defects. The numbers in the figure represent the numbers of plates (cohorts) of 10 animals each. **b** Behavioral screening of candidate mutants based on turn defects. The numbers in the figure represent the numbers of plates (cohorts) of 10 $\pm$ 3 animals each. The error bars in these figures represent the $\pm$ SEM values.

Supplementary Figure 6

S Hori and S Mitani

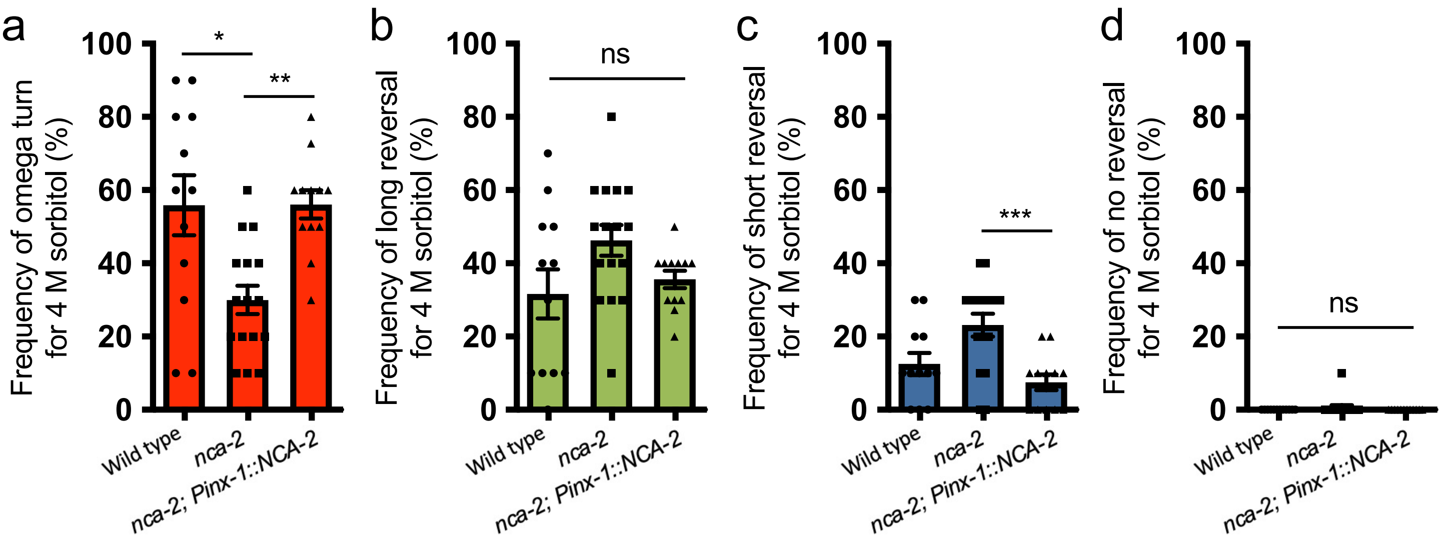

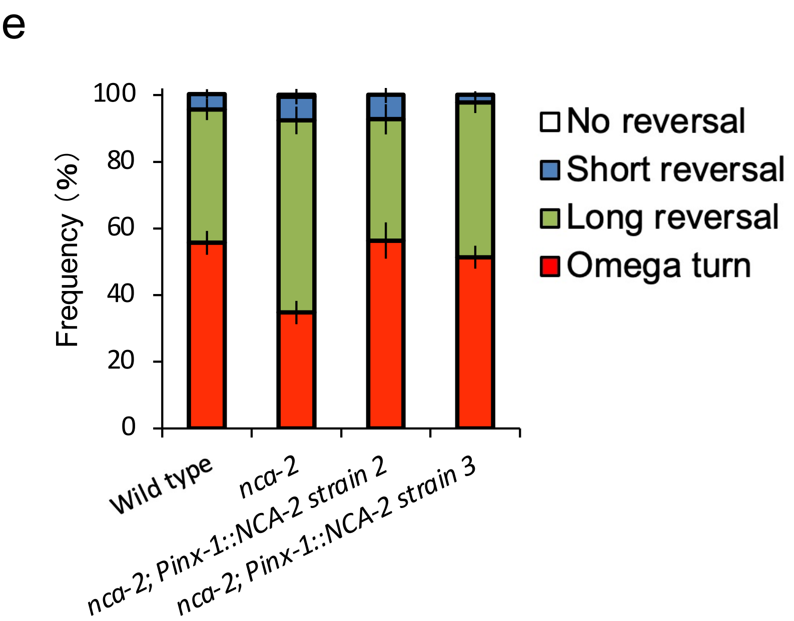

Supplementary Fig. S7 Scatterplots of each behavioral frequency across all replicates (mean ± SEM indicated), related for Fig. 3e. a-d Omega turn, long reversal, short reversal and no reversal rates related for Fig. 3e, respectively. **e** Results of two additional lines for P*inx-1*::NCA-2 rescue. Both a strain 2 (*tm1305;jskEx5628*) and a strain 3 (*tm1305;jskEx0001*) rescued *nca-2* behavioral phenotypes (n = 30, 21, 11, 22). **f-i** Omega turn, long reversal, short reversal and no reversal rates related for Fig. 1h, respectively. ***indicates $P < 0.001$, **indicates $P < 0.01$, *indicates $P < 0.05$, ns indicates $P > 0.05$ (one-way ANOVA followed by Tukey's post hoc test). The error bars in this figure represent the ± SEM values.

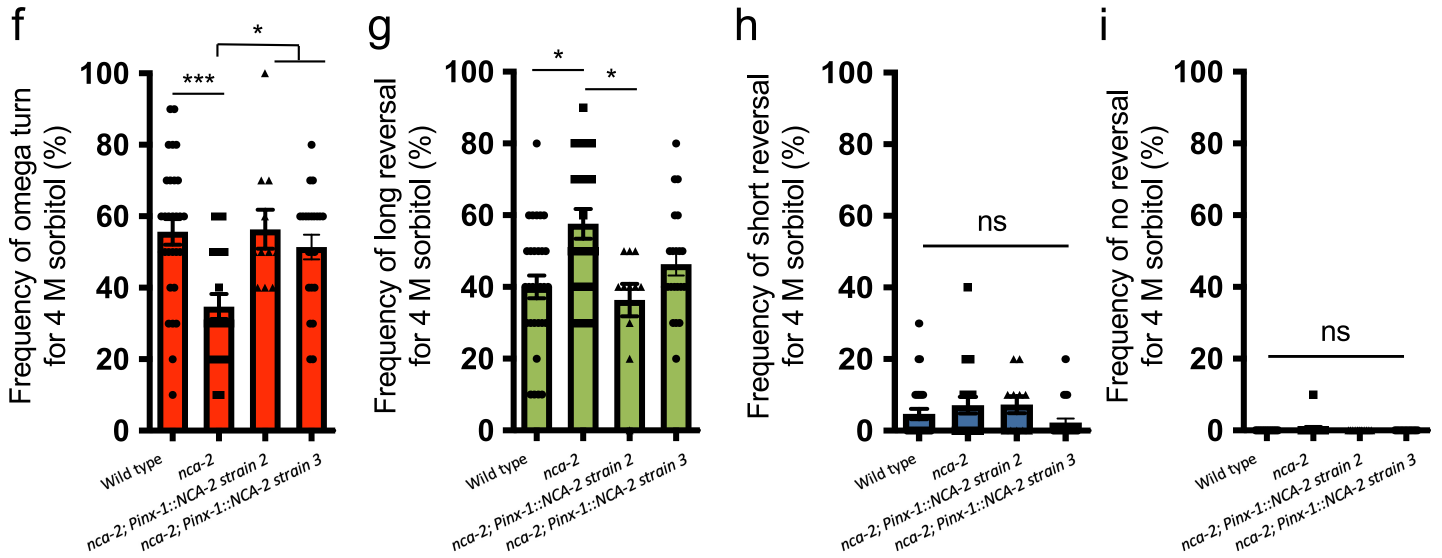

Supplementary Figure 7

S Hori and S Mitani

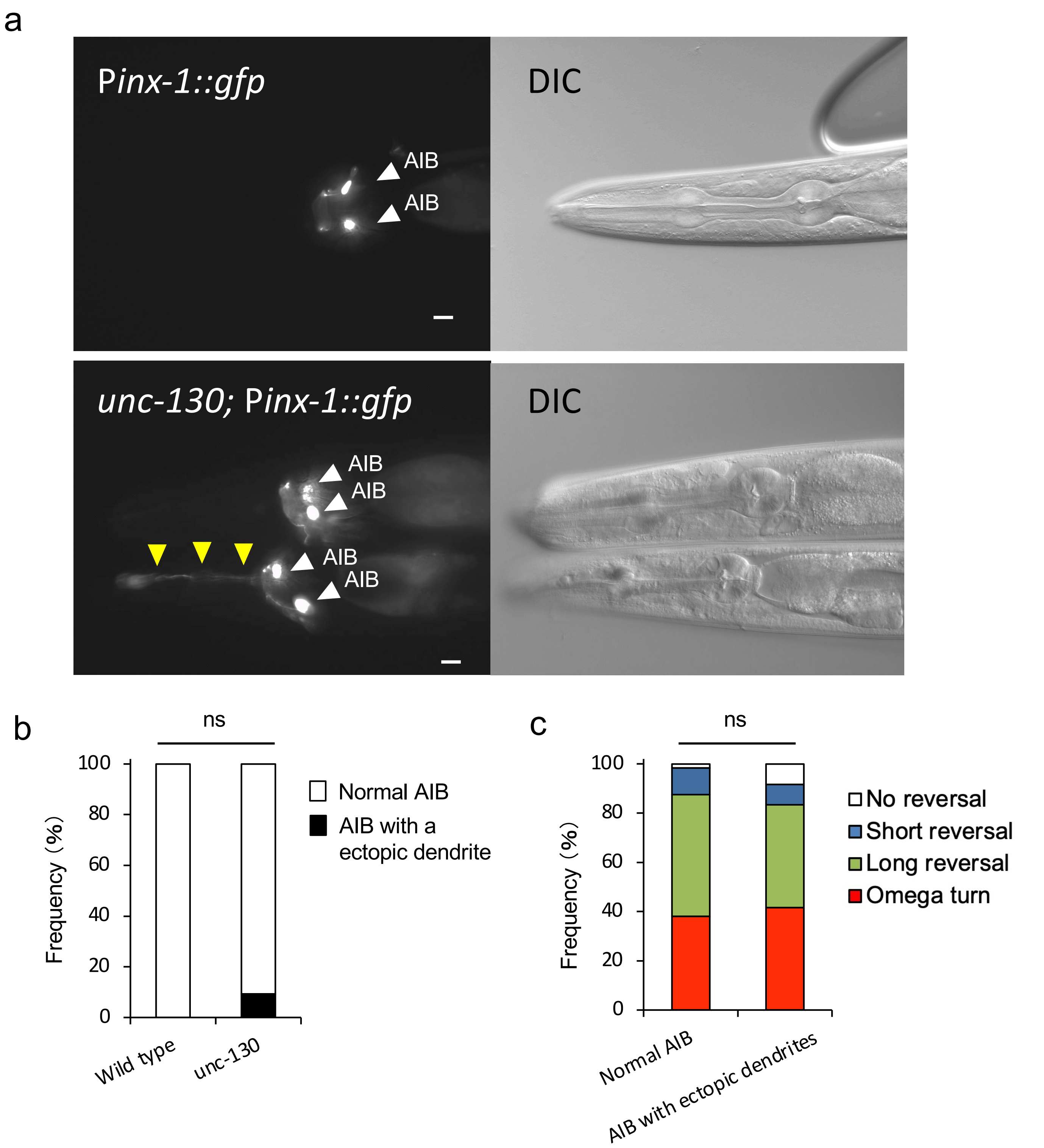

Supplementary Fig. S8 Detailed analysis of *inx-1*-marker positive cells. a GFP-positive cells observed in a wild-type background animal and an *unc-130* animal (AIB neurons: white arrowheads), and an ectopic dendrite in an *unc-130* animal (yellow arrowhead). **b** Ectopic dendrites are infrequently observed. AIB with ectopic neurites was 9.38% (n = 12 animals) in the *unc-130* mutants, but there was no significant difference between wild-type animals and *unc-130* mutants (n = 40, 128 animals). **c** No correlation between the presence of ectopic neurites and turn/reversal frequency (P = 0.892, n = 40, 128 animals, Fisher's exact test).

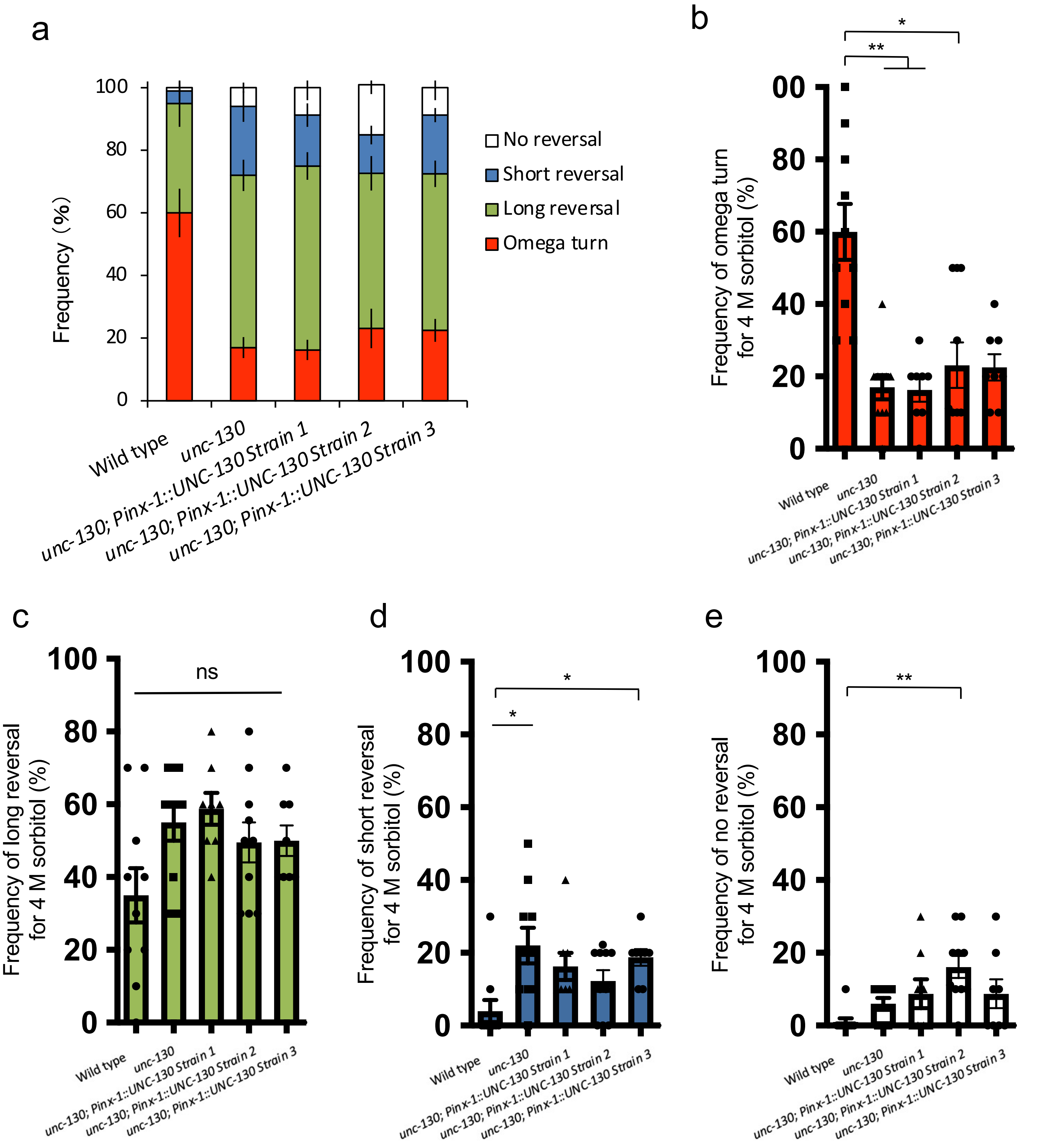

Supplementary Fig. S9 Results of P*inx-1*::UNC-130 rescue. a Three strains (*tm320;jskEx0024, tm320;jskEx0028* and *tm320;jskEx0029*) did not rescue *unc-130* behavioral phenotypes (n = 10, 10, 8, 10, 8)**. b-e** Scatterplots of each behavioral frequency across all replicates (mean ± SEM indicated). Omega turn, long reversal, short reversal and no reversal rates, respectively. **indicates $P < 0.01$, *indicates $P < 0.05$, ns indicates $P > 0.05$ (one-way ANOVA followed by Tukey's post hoc test). The error bars in this figure represent the ± SEM values.

Supplementary Figure 9

S Hori and S Mitani

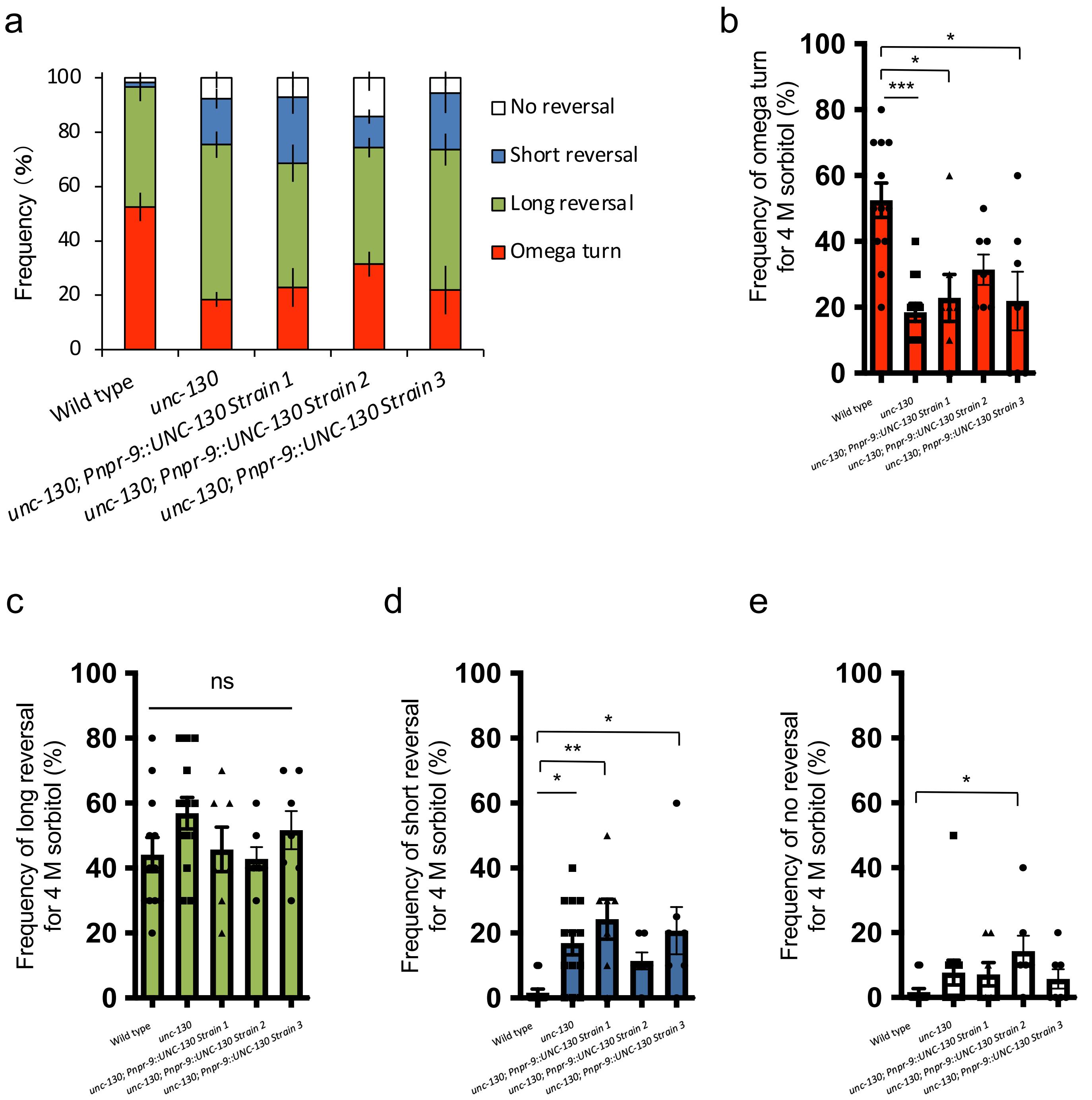

Supplementary Fig. S10 Results of P*npr-9*::UNC-130 rescue. a Three strains (*tm320;jskEx0011*, *tm320;jskEx0013* and *tm320;jskEx0014*) did not rescue *unc-130* behavioral phenotypes (n = 16, 18, 14, 10, 10)**. b-e** Scatterplots of each behavioral frequency across all replicates (mean ± SEM indicated). Omega turn, long reversal, short reversal and no reversal rates, respectively. ***indicates P < 0.001, *indicates P < 0.05, ns indicates P > 0.05 (one-way ANOVA followed by Tukey's post hoc test). The error bars in this figure represent the ± SEM values.

Supplementary Figure 10

S Hori and S Mitani

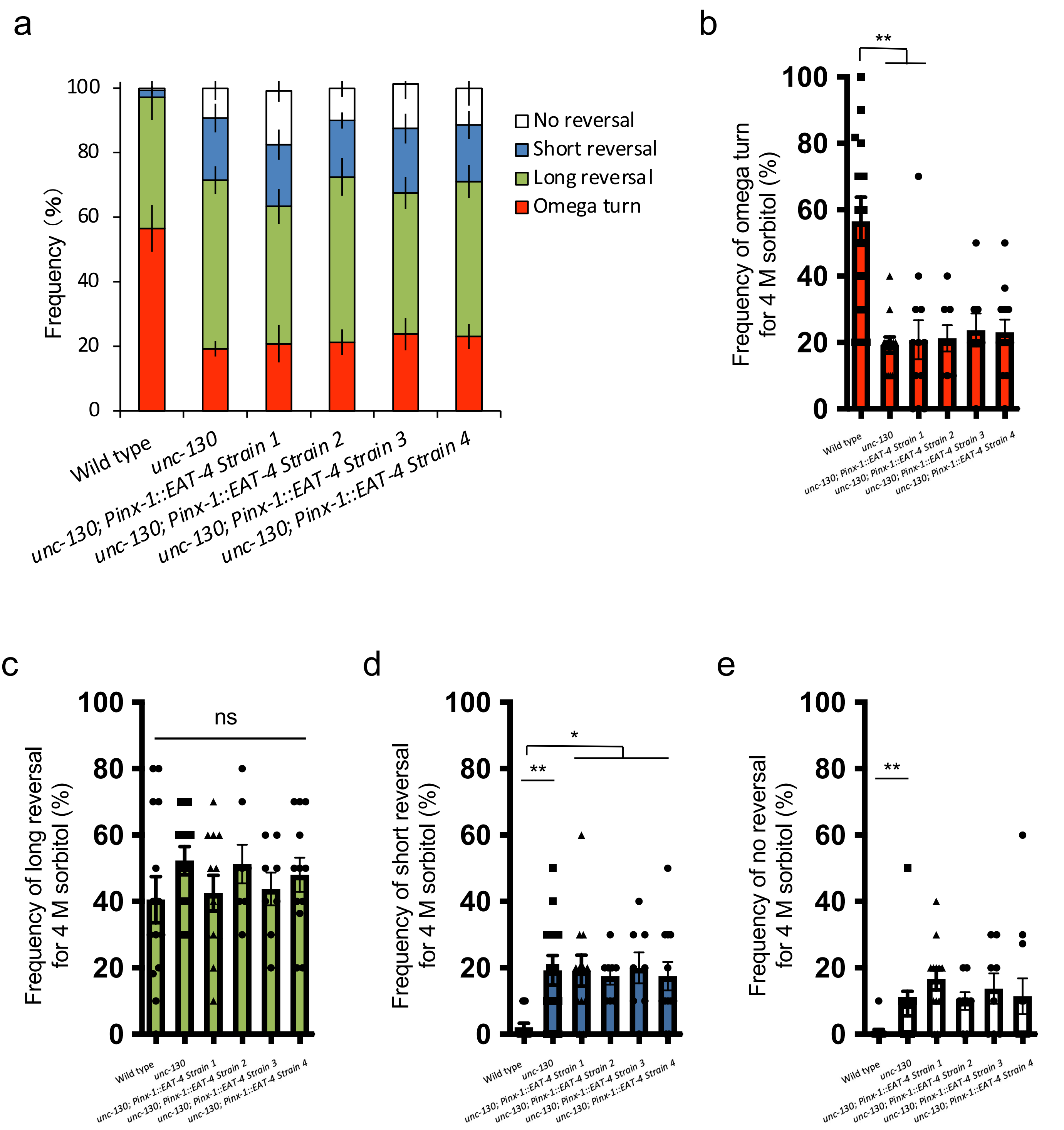

Supplementary Fig. S11 Results of P*inx-1*::EAT-4 rescue. a Three strains (*tm320;jskEx0019, tm320;jskEx0020* and *tm320;jskEx0021*) did not rescue *unc-130* behavioral phenotypes (n = 13, 12, 8, 8, 12). **b-e** Scatterplots of each behavioral frequency across all replicates (mean ± SEM indicated). Omega turn, long reversal, short reversal and no reversal rates, respectively. ***indicates P < 0.001, *indicates P < 0.05, ns indicates P > 0.05 (one-way ANOVA followed by Tukey's post hoc test). The error bars in this figure represent the ± SEM values.

Supplementary Figure 11

S Hori and S Mitani

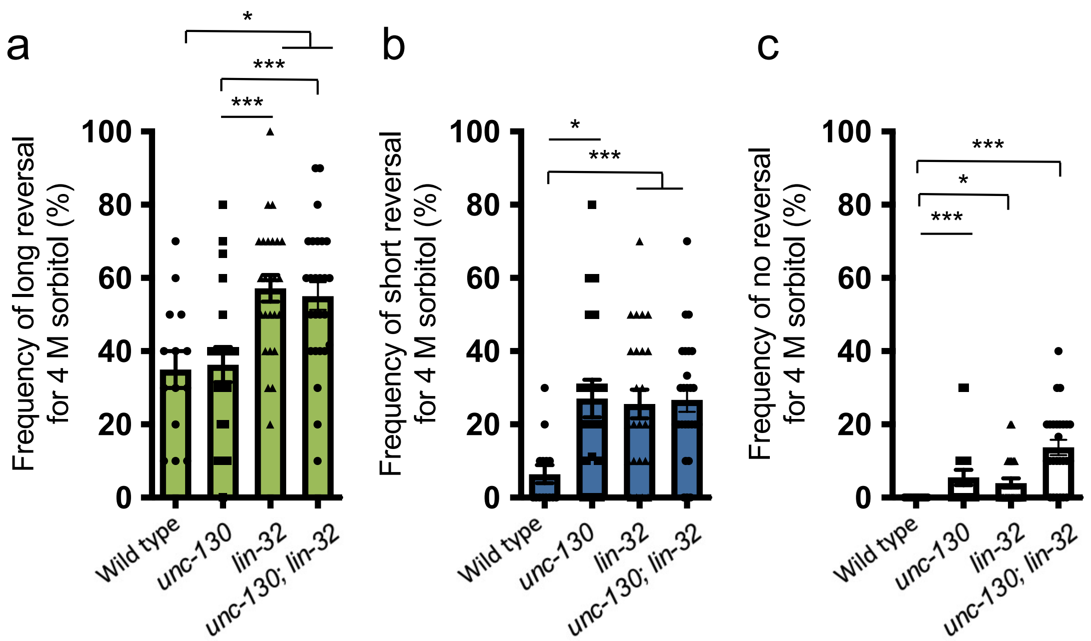

Supplementary Fig. S12 Scatterplots of each behavioral frequency across all replicates (mean ± SEM indicated), related for Fig. 5c. a-c Scatterplots of each behavioral frequency across all replicates (mean ± SEM indicated). Omega turn, long reversal, short reversal and no reversal rates, respectively. ***indicates $P < 0.001$, **indicates $P < 0.01$, *indicates $P < 0.05$, ns indicates $P > 0.05$ (one-way ANOVA followed by Tukey's post hoc test). The error bars in this figure represent the ± SEM values.

S Hori and S Mitani

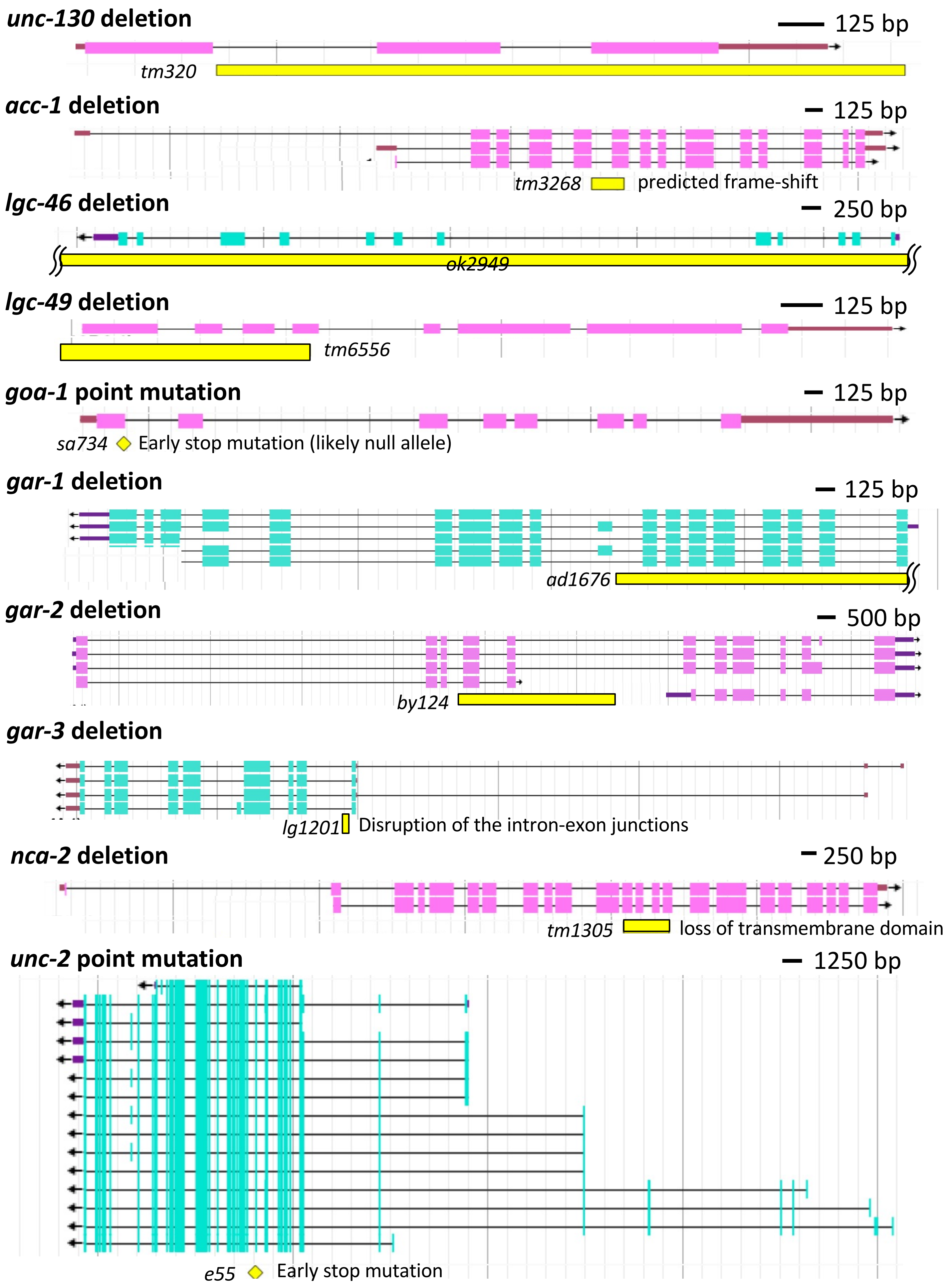

Supplementary Figure 13-1

S Hori and S Mitani

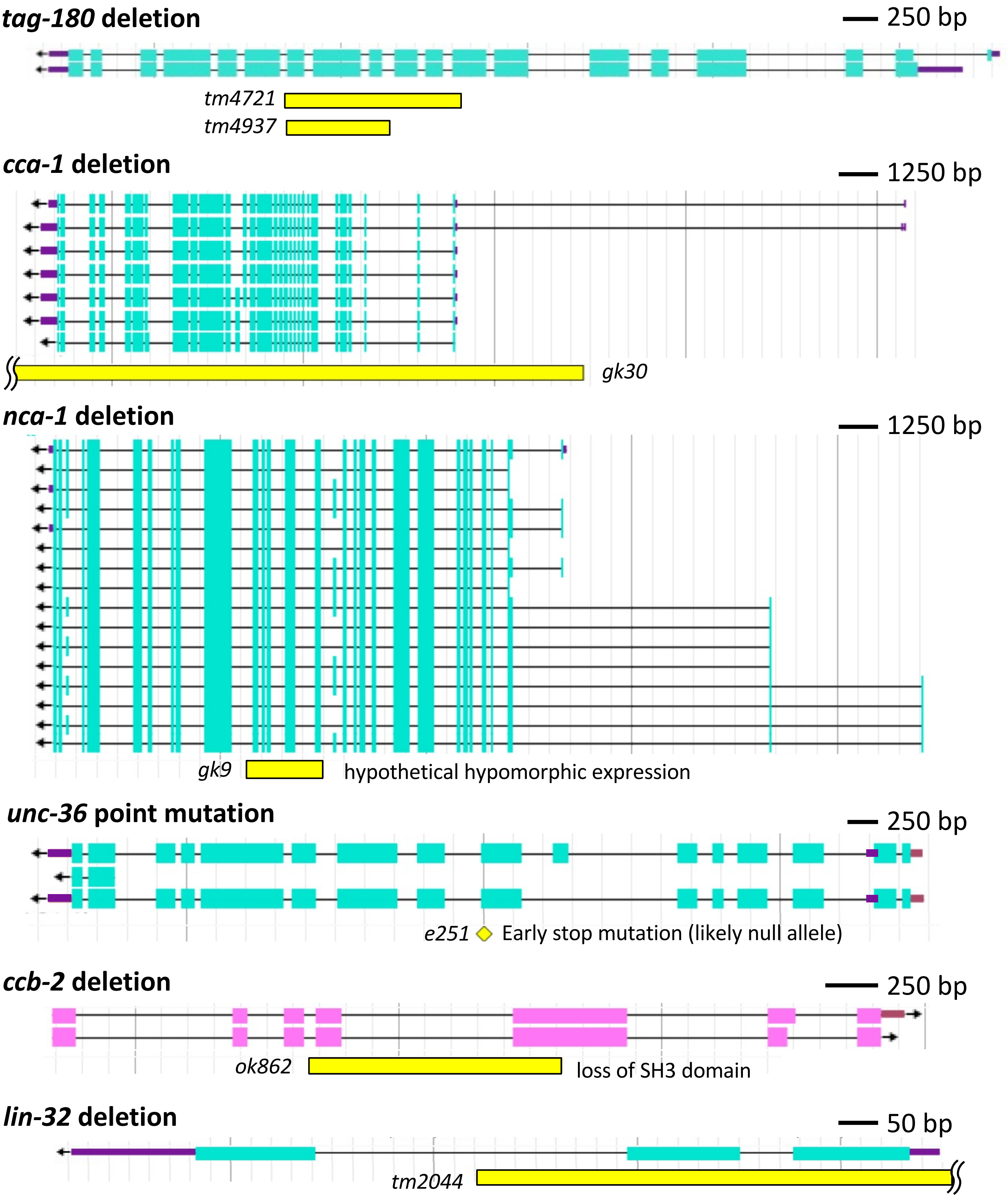

Supplementary Fig. S13 Gene structures of the mutants. UTR, exon, and intron structures of each gene. For genes with subtypes, all structures are drawn. Magenta, light pink, and gray represent the UTRs, exons, and introns in genes encoded in the (+) strand of the chromosome, respectively. Purple, blue, and gray represent the UTRs, the exons, and the introns in genes encoded in the (-) strand of the chromosome, respectively. Yellow bars and rhombi illustrate the sites of deletion and point mutation, respectively. The predicted results of small deletion or point mutations are noted. The error bars in this figure represent the ± SEM values.
